# Functional signatures of evolutionarily young CTCF binding sites

**DOI:** 10.1186/s12915-020-00863-8

**Published:** 2020-09-23

**Authors:** Dhoyazan Azazi, Jonathan M. Mudge, Duncan T. Odom, Paul Flicek

**Affiliations:** 1 European Molecular Biology Laboratory, European Bioinformatics Institute, Wellcome Genome Campus, Hinxton, Cambridge, CB10 1SD UK; 2 University of Cambridge, Cancer Research UK Cambridge Institute, Robinson Way, Cambridge, CB2 0RE UK; 3 German Cancer Research Center (DKFZ), Division Regulatory Genomics and Cancer Evolution, 69120 Heidelberg, Germany; 4 Wellcome Sanger Institute, Wellcome Genome Campus, Hinxton, Cambridge, CB10 1SA UK

**Keywords:** CTCF, Gene regulation, Evolutionary genomics

## Abstract

**Background:**

The introduction of novel CTCF binding sites in gene regulatory regions in the rodent lineage is partly the effect of transposable element expansion, particularly in the murine lineage. The exact mechanism and functional impact of evolutionarily novel CTCF binding sites are not yet fully understood. We investigated the impact of novel subspecies-specific CTCF binding sites in two *Mus* genus subspecies, *Mus musculus domesticus* and *Mus musculus castaneus*, that diverged 0.5 million years ago.

**Results:**

CTCF binding site evolution is influenced by the action of the B2-B4 family of transposable elements independently in both lineages, leading to the proliferation of novel CTCF binding sites. A subset of evolutionarily young sites may harbour transcriptional functionality as evidenced by the stability of their binding across multiple tissues in *M. musculus domesticus* (BL6), while overall the distance of subspecies-specific CTCF binding to the nearest transcription start sites and/or topologically associated domains (TADs) is largely similar to *musculus*-common CTCF sites. Remarkably, we discovered a recurrent regulatory architecture consisting of a CTCF binding site and an interferon gene that appears to have been tandemly duplicated to create a 15-gene cluster on chromosome 4, thus forming a novel BL6 specific immune locus in which CTCF may play a regulatory role.

**Conclusions:**

Our results demonstrate that thousands of CTCF binding sites show multiple functional signatures rapidly after incorporation into the genome.

## Background

Genetic differences within and between species predominantly lie in the noncoding sequence of the regulatory regions of the genome, whose function and significance largely remain poorly understood [1–3]. While interspecies comparisons of mammalian genomes have revealed that protein-coding genes have been subject to strong selective pressures [4], tissue-specific transcription factor binding diverges more frequently between species [5–8].

CCCTC-binding factor (CTCF) is a ubiquitously expressed 11 zinc-finger master genome organiser [9] shared between all vertebrates [10]. It plays a part in many basic cellular roles including transcriptional activation and repression [11, 12], X-inactivation [13], establishing 3D genome architecture [14], enhancer insulation [15], and alternative splicing [16]. The importance of these functions is illustrated by CTCF knockout being embryonic lethal [17] and tissue-specific conditional knockouts having dramatic developmental abnormalities [18, 19]. The CTCF protein itself shows a remarkable degree of evolutionary conservation, with 93% amino acid identity of the full protein sequence between human and chicken and 100% identity in its DNA binding domain [20, 21].

One of the most important functions of CTCF is to help establish 3D genome structure through interaction with the cohesin complex [22–25]. The colocalised binding of CTCF and the cohesin complex can create chromatin loops, demarcated by two CTCF molecules bound to the genome and stabilised by cohesin [26–28]. This gives rise to topologically associating domains (TADs) [13], demarcated by CTCF sites deeply conserved across mammals [29], and with mostly invariant positions across species and tissues [30, 31].

Many changes in the regulatory non-coding genome between species are the consequences of the co-option of repetitive sequences for active binding of transcription factors [32–38]. Across mammals, the evolution of CTCF binding has been driven by repeated waves of expansions of transposable elements that deposited its binding motif in novel genomic locations [35–37]. Specifically, within the Murine lineage, CTCF binding motifs have recently been spread through the short interspersed nuclear element (SINE) family of transposable elements. Similar repeat-driven transcription factor binding site birth expansions have been observed for other tissue-specific transcription factors in stem cells [34] and in pregnancy-associated tissues [39], suggesting that repeat expansions are a common mechanism used to remodel mammalian genomes [32]. However, the potential functional roles of very young insertions of transcription factor binding sites through repeats and their genomic characteristics have not yet been well characterised.

**Fig. 1:**
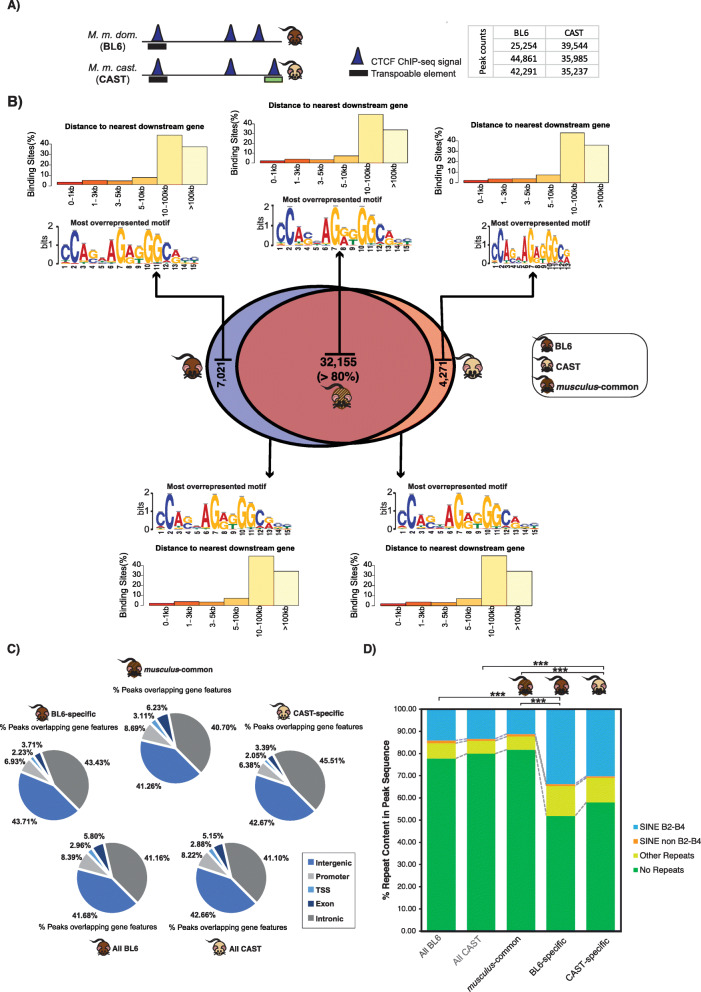
Overview of genomics features and evolutionary conservation of CTCF binding in the BL6 and CAST subspecies. **a** A schematic example of the contribution of transposable elements novel subspecies-specific CTCF binding. The peaks represent CTCF binding as determined from ChIP-seq experiments, while the boxes denote different groups of transposable elements (black = SINE, green = LTR). The table shows the peak counts (binding sites) retrieved from the three biological replicates for each subspecies. All downstream analysis utilised peaks common to a minimum of two replicates. **b** The Venn diagram shows the degree of CTCF binding overlap in whole genome alignments between the *Mus musculus domesticus* (BL6) and *Mus musculus castaneus* (CAST) mouse subspecies. CTCF binding sites found aligned in orthologous locations are called *musculus*-common, while those with no alignment in the other species are subspecies-specific. For each evolutionary class of CTCF sites (above Venn diagram) and for all sites regardless of conservation between species (below Venn diagram), the most represented sequence motif and the distance to the nearest downstream genes. **c** The pie charts show the gene features overlapping CTCF sites for all evolutionary classes in the Venn diagram in **b**. **d** The repeat content of all CTCF binding sites, and each evolutionary category described in **b** is measured as the percentage of a CTCF binding sites’ sequence that overlaps a repeat element. The asterisks indicate the significance of enrichment of SINE B2-B4 elements between subspecies-specific sets and all CTCF binding sites for both species and the *musculus*-common set (binomial tests, ***p < 0.0001)

Leveraging the availability of high-quality genome sequences from laboratory strains and species within the *Mus* genus created by the Mouse Genomes Project [40–43], we illustrate how repetitive elements have remodelled CTCF binding in two *Mus* genus subspecies sharing a common ancestor 0.5 million years ago (MYA): *Mus musculus domesticus* (C57BL/6J or BL6) and *Mus musculus castaneus* (CAST) (Fig. 1a). We found that almost half of the subspecies-specific CTCF binding sites are from repeat origin but have signatures of function and genomic occupancy patterns that are largely similar to CTCF sites common between the subspecies. We next identified a subset of these subspecies-specific sites that was bound in multiple tissues and exhibit heightened recruitment of cohesin-complex subunits, suggesting active participation in loop formation and higher functionality of these sites. We also found a cluster of interferon genes with BL6 subspecies-specific CTCF and cohesin colocalised binding sites on mouse chromosome 4 that apparently arose via a recent tandem duplication event. Taken together, these results demonstrate the pace at which evolutionarily young CTCF binding sites appear in the genome and acquire functional signatures.

## Results

To study the evolution of CTCF binding between closely related species, we performed chromatin immunoprecipitation followed by high-throughput sequencing (ChIP-seq) in liver samples from two mouse subspecies: BL6 and CAST (Methods). We used three biological replicates for each subspecies and retained only those peaks present in at least two individuals, yielding comparable numbers of CTCF binding sites in both subspecies (Fig. 1a). We next performed evolutionary analysis of CTCF binding by finding those sites that occurred in orthologous locations in a pairwise alignment between the BL6 and CAST genomes. We found that the vast majority (> 32,000) of CTCF binding occurs in orthologous locations, which we refer to as *musculus*-common sites (Fig. 1b), in line with previous studies across more diverged mammals [29] and rodents [44]. However, even within these closely related subspecies, a considerable amount of biologically reproducible CTCF binding was found at subspecies-specific locations. In total, we identified in excess of 11,000 subspecies-specific CTCF binding sites in BL6 and CAST.

We next examined the genomic characteristics of subspecies-specific and *musculus*-common CTCF binding sites. Analysis of binding site positions relative to transcription start sites (TSSs) revealed an almost identical genomic location distribution, regardless of evolutionary conservation, with the largest portion of CTCF binding more than 10 kb from the nearest TSS (Fig. 1b). We then classified each CTCF binding site by the genomic feature it overlaps (Fig. 1b) and again found little differences between different categories of conservation. As expected, around 41–43% of CTCF binding within both *musculus*-common and subspecies-specific sites is intergenic, and the rest occurs within promoters or genes [45]. Similarly, the canonical CTCF binding motif was retrieved from all CTCF binding sites (Fig. 1c). Analyses of the genomic features of *musculus*-common and subspecies-specific CTCF binding suggests that the recently evolved sites perform similar functions to conserved sites.

### Transposable elements are responsible for half of subspecies-specific binding

Muridae lineage species/subspecies, and these two subspecies in particular, have similar genomic repeat profiles [42, 46]. Given the known contribution of transposable elements to CTCF binding site evolution [32, 35], we quantified the transposable element content in CTCF binding sites. Both subspecies had comparable overall repeat profiles with approximately 21% of all CTCF binding occurring within a repetitive element (Fig. 1d), mostly of the B2-B4 rodent-specific subfamily of SINEs. However, for both BL6 and CAST, subspecies-specific binding sites were significantly more enriched in repetitive elements than *musculus*-common sites. Specifically, 34% of subspecies-specific CTCF binding overlapped a transposable element of the B2-B4 subfamily, more than a twofold increase compared to all sites overlapping B2-B4 (14%) in both BL6-specific (BL6-specific vs. All BL6: binomial test *p* value = 3.1E−07; BL6-specific vs. *musculus*-common: binomial test *p* value = 9.0E−10) and CAST-specific binding sites (CAST-specific vs. All CAST: binomial test *p* value = 7.4E−06; CAST-specific vs. musculus-common: binomial test *p* value = 1.9E−07) (Fig. 1d). The contribution of the SINE B2-B4 subfamily to subspecies-specific CTCF binding sites is likely an underestimation; an additional 15% of sites identified in only one biological replicate overlap with SINE B2-B4 and most likely represent CTCF binding that is weaker and/or variable between individuals.

**Fig. 2:**
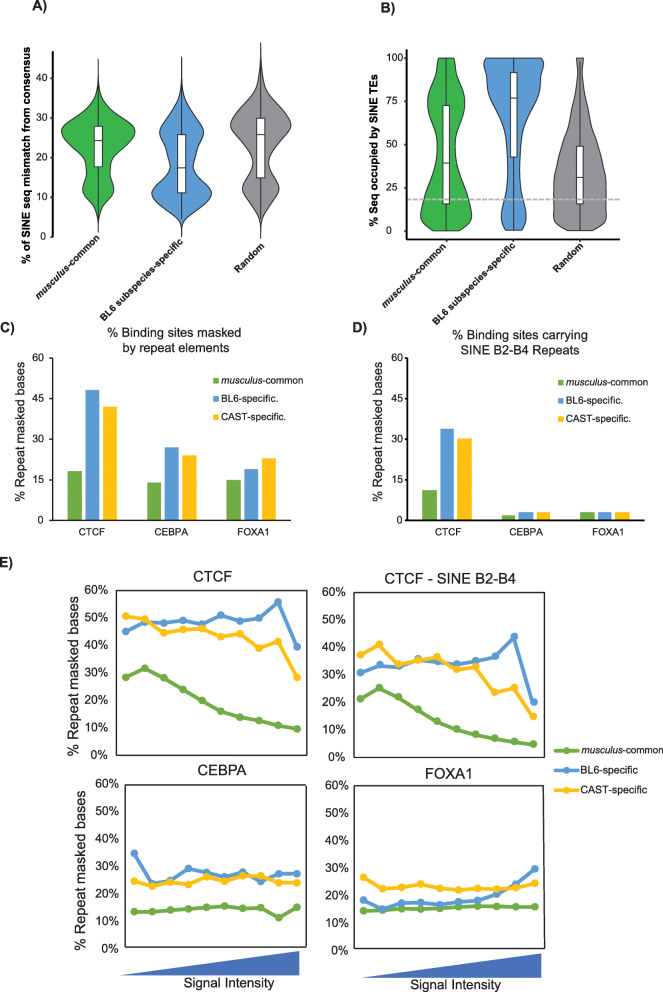
SINE transposable elements drive CTCF subspecies-specific binding. **a** The proportion of sequence mismatches/substitution from consensus in SINE TEs for all TE superfamilies in the different evolutionary categories of CTCF sites, compared to a matched random set. **b** The proportion of sequence masked by SINE repeat elements in conserved/subspecies-specific CTCF sites, compared to a matched random set. The boxplots within each violin plot show the variation in extent of sequence masking for each category. The dashed grey line denotes the average genomic sequence occupied by SINE TEs in all TE-masked sequences of the mouse genome. **c** Comparison of the total repeat element content in CTCF, CEBPA and FOXA1 transcription factor binding sites between sites with orthologous binding in the other subspecies (*musculus*-common) and subspecies-specific sites (BL6- and CAST-specific). All differences between subspecies-specific binding enrichment in repeat content with either the repeat content of all TF sites or the *musculus*-common set where found to be statistically significant (Binomial test, all *p* values < 2.2e−16). **d** Comparison of the total content of the SINE B2-B4 transposable element subfamily in CTCF, CEBPA and FOXA1 transcription factor binding sites between sites with orthologous binding in the other subspecies (*musculus*-common) and subspecies-specific sites (BL6- and CAST-specific). **e** The dependence of repeat element content of binding sites on signal strength for all CTCF binding sites, the subset of CTCF sites within SINE B2-B4 elements, CEBPA and FOXA1 transcription factors. Within each transcription factor set, the data is binned in 10% bins based on binding site signal strength as estimated from the number of ChIP-seq reads mapped

We compared the occurrence of *musculus*-common and BL6-specific CTCF binding sites to the overall distribution of the four most common TE superfamilies in the mouse genome (Additional file 1: Figure S1A) and found that CTCF sites were highly enriched with SINE TEs: 71% for *musculus*-common sites, rising up to 76% for BL6-specific sites, compared to 20% in the whole genome (*χ*^2^ test, *p* value < 2.2e−16). Within the SINE superfamily both types of binding sites overlapped almost exclusively the B2-B4 family (Additional file 1: Figure S1B). Most of these BL6-specific sites are younger, as estimated by the lower levels of sequence mismatch from the consensus sequence (median = 16% for BL6-specific versus 22% and 24% for *musculus*-common and randomised genomic regions respectively, Mann-Whitney *U* test, both *p* value < 2.2e−16) (Fig. 2a for SINE repeats and S1C for all superfamilies of TEs). Not only do BL6-specific CTCF bind in SINE TEs more often than their *musculus*-common counterparts, but also for each BL6-specific binding site embedded in a SINE TE, more of the sequence is SINE-derived (Fig. 2b). Specifically, 50% of the BL6-specific sequences were 77% masked by SINE repeats, compared to *musculus*-common site (median = 39%) and randomised regions (median = 31%) (Mann-Whitney *U* test, both *p* value < 2.2e−16). This recent cluster of SINE-derived CTCF sites suggests that a post-divergence expansion of the binding sites continued in each mouse lineage separately and may yet still be ongoing.

To investigate how repeat element expansion contributed to the binding of other transcription factors expressed in the liver, we performed analogous evolutionary and repeat content analyses for the liver-specific transcription factors CEBPA and FOXA1. We first reanalysed publicly available ChIP-seq experiments [47] to identify *musculus*-common and subspecies-specific binding in BL6 and CAST as described above for CTCF (Methods). In both subspecies and for both liver-specific transcription factors, repeat elements contributed more to subspecies-specific than *musculus-*common binding, but to a much smaller extent than for CTCF (Fig. 2c). Unlike CTCF binding, there was negligible overlap of CEBPA or FOXA1 with SINE B2-B4 elements (Figs. 2d and S1D) and no discernible pattern of overlap with any other specific transposable element group. These results further confirm earlier studies that show SINE B2-B4 elements are specialised for the expansion of CTCF binding sites and contribute to a larger portion of subspecies-specific binding than tissue-specific transcription factors.

We next investigated the relationship between the age of transposable element insertion, as estimated by the repeat content within peaks, and transcription factor binding strength for all three transcription factors. We measured the repeat content of binding sites at increasing experimentally determined ChIP-seq signal intensities (Methods) and found their genomic distribution to be indistinguishable from those CTCF binding sites with higher intensity (Fig. 1b). In the *musculus*-common set of CTCF binding sites, the repeat content noticeably drops at increased binding intensities (Fig. 2e) suggesting that older transposable elements have higher affinity for CTCF binding. In contrast, in the subspecies-specific sets, the overall repeat content and the SINE B2-B4 content were comparable across all ChIP-seq signal intensities (Fig. 2e). The repeat content of the tissue-specific transcription factors CEBPA and FOXA1 was also independent from ChIP-seq signal intensity and similar across both *musculus*-common and subspecies-specific sites (Fig. 2e). Interestingly, most tissue-specific transcription factor binding and *musculus-*common CTCF binding occurred in sites with noticeably smaller repeat content than subspecies-specific CTCF binding. Their strong binding affinity suggests that functional subspecies-specific CTCF binding sites have arisen from transposable elements more recently than tissue-specific transcription factors.

Taken together, these results demonstrate the extent and speed at which transposable elements can shape transcription factor binding. In just one million years of evolutionary time separating BL6 and CAST transposable elements have apparently contributed to almost half of the subspecies-specific CTCF binding profiles.

### Subspecies-specific CTCF binding is predominantly tissue-restricted

**Fig. 3:**
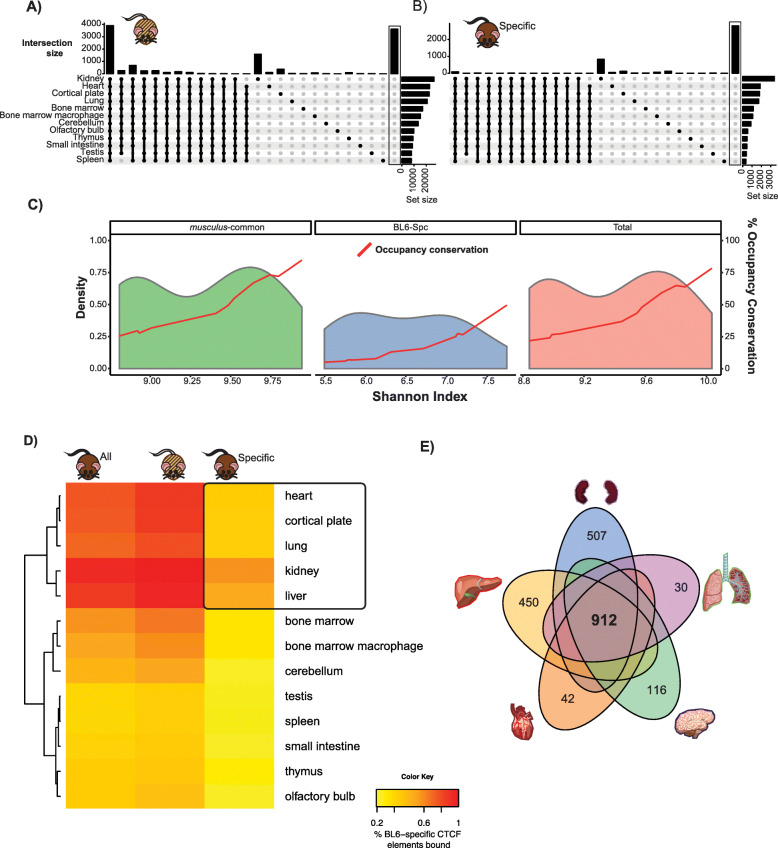
Almost 10,000 BL6 subspecies-specific CTCF binding sites are shared among five tissues. **a, b** UpSet plot of CTCF binding sites we mapped in liver and their binding across 12 adult tissues as identified in the mouse ENCODE project [48] for *musculus*-common (**a**) and BL6-specific (**b**) sites. The number of sites bound at each combination of tissues is indicated on the *y*-axis on the top bar chart. The rightmost bar on each UpSet plot (*boxed*) indicates the number of CTCF binding sites that were not found to be bound in any other ENCODE tissue library. **c** Strong association between tissue-wide CTCF binding and occupancy conservation across 12 tissues. The plots show the density of CTCF binding sites across different tissue diversity values. The diversity values indicated on the *x*-axis were calculated using the Shannon Diversity Index (see the “Methods” section). The red line is for the proportion of conserved CTCF occupancy within each bin of Shannon index, calculated based on the number of CTCF sites bound for each category across tissues separately. **d** Tissue distribution analysis of CTCF binding sites found to be *musculus*-common and subspecies-specific using ENCODE CTCF ChIP-seq data across 13 tissues. The heatmap indicates the overlap of *musculus*-common/BL6-specific CTCF binding which also binds in ENCODE tissues, with the five most similar tissues for BL6-specific binding highlighted. **e** Venn diagram highlighting CTCF BL6-specific sites that are shared between all of the five most similar tissues and those found in only one of the tissues

Although CTCF binding is more consistent across tissues than most other transcription factors, many binding sites are tissue-specific [48–50]. To investigate the relationship between subspecies-specific binding and tissue-specificity, we determined the tissue distribution of binding sites across adult mouse tissues. We reanalysed ENCODE CTCF ChIP-seq data for BL6 adult male mice from 12 tissues: lung, bone marrow, bone marrow macrophages, cortical plate, cerebellum, heart, kidney, thymus, spleen, olfactory bulb, small intestine and testis [48]. Analysis of ENCODE CTCF libraries showed that almost 4000 sites (12.59%) are bound in all ENCODE tissues, and an additional 1800 sites have their occupancy conserved in a minimum of 11 tissues. More than 6000 of all CTCF sites we identified appear to be liver-specific (Additional file 2: Figure S2A) which might partly be due to different experimental protocols and sequencing depth. Since CTCF binding is not tissue-specific, the number of sites across tissues should be comparable [30, 31]. This discrepancy would potentially be resolved with better libraries for the other tissues that yield a higher number of CTCF peaks so that we may be able to identify more of these BL6-specific sites shared in other tissues. When stratified based on their evolutionary origin, the patterns above were similar in the *musculus*-common set of CTCF binding sites; 98% (> 3900) of all CTCF sites bound in all 12 tissues were *musculus*-common (Fig. 3a). On the other hand, slightly over 1% of all BL6-specific CTCF sites were bound in all 12 tissues, and 41% of these sites (> 2800) were found only in the liver (Fig. 3b).

We next investigated the relationship between CTCF binding conservation and tissue distribution across evolutionary classes of binding sites. We explored the tissue distribution of CTCF binding across all of the 12 mouse ENCODE tissues using the Shannon Index [51] which quantifies the abundance and enrichment of binding sites (Methods) and correlated these with occupancy conservation. The results matched the findings above, showing high Shannon index values across tissues to be correlated with a greater degree of CTCF occupancy conservation (Fig. 3c, rightmost panel). These trends were strongest in *musculus*-common sites, with overall higher density at higher values of the diversity index (Fig. 3c, leftmost panel). The diversity index values for the BL6-specific CTCF sites were much lower—a further sign that subspecies-specific CTCF binding is predominantly tissue-specific—while its occupancy in other tissues is far more restricted.

We next compared the extent of tissue-shared binding between BL6-specific and *musculus-*common CTCF binding sites. While 50% of all *musculus*-common CTCF sites are bound in more than five tissues, only 16% of subspecies-specific sites bind in a minimum of five tissues (Additional file 2: Figure S2B). The number of BL6-specific sites identified in multiple tissues was lower than would be expected based on their overall constitution of the total set of sites, except for the sites identified in 3 tissues (chi-square with Bonferroni multiple testing correction, all *p* values < 2.2e−16, except for 3 tissues where *p* value = 0.0083). We further calculated the extent of CTCF binding overlap between specific ENCODE tissues and found that a substantial subset of both *musculus*-common and BL6-specific sites are bound in multiple other tissues (Fig. 3d). BL6-specific sites were underrepresented for each tissue compared to their overall genomic distribution (chi-square test with Bonferroni multiple testing correction, all *p* values < 2.2e−16). Almost all BL6-specific binding sites we identified in the liver are also present in the independent ENCODE liver experiments, demonstrating that there is very little inter-individual (i.e. between biological replicates) variation for these sites. We selected the top five ENCODE tissues by the number of shared CTCF binding with our BL6-specific set for further analysis (Fig. 3e). As expected, ENCODE liver has the most overlap with our liver datasets and kidney has the next most similar binding profile. For the BL6 binding sites, we identified as *musculus*-common 67–85% are bound in these five tissues, compared to only 26–49% of the BL6-specific sites. The CTCF binding sites shared across at least one of the five tissues constitute 13% of all of BL6-specific CTCF sites, with only 4% (912) being bound in all tissues. Thus, these results show that subspecies-specific CTCF binding has relatively little variation between biological replicates and is more tissue-restricted than *musculus*-common sites.

### Regulatory signatures and functional impact of subspecies-specific CTCF occupancy

**Fig. 4:**
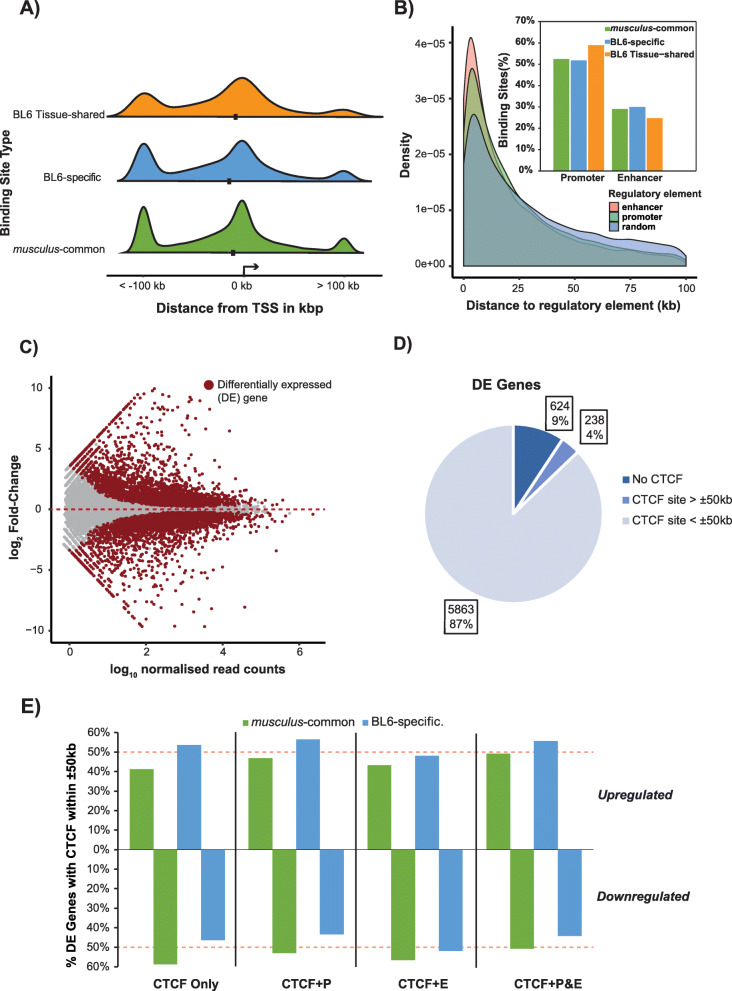
Regulatory signatures of BL6-specific CTCF binding. **a** Distribution of the distances of CTCF binding sites to the transcription start site (TSS) of the nearest downstream gene based on their evolutionary class and tissue-distribution. The median is marked with a black point. **b** The distance from *musculus*-common and BL6-specific CTCF sites to their nearest non-overlapping active regulatory region is shorter than that of random genomic regions. The bar chart inset shows the type of regulatory element that is closest (but non-overlapping) to *musculus*-common and BL6-specific CTCF sites. **c** Over 6000 1-to-1 orthologous genes are differentially expressed (DE) between BL6 and CAST. Significant DE genes (FDR < 0.05) are highlighted against background of non-DE genes (*grey*) [29]. **d** CTCF binding is observed within a 50-kb distance of the vast majority of DE genes. **e** BL6-specific CTCF binding associates more with downregulated DE genes than *musculus*-common CTCF binding, either alone or with other associated active *cis*-regulatory elements. The bar plots display the proportions of DE genes associated with a CTCF binding sites within 50 kb, with an active promoter, an enhancer, and both

To gain insight into the possible functional roles of evolutionarily distinct CTCF binding classes, we examined the genomic features near *musculus*-common CTCF sites and subspecies-specific sites that were either tissue-restricted or tissue-shared. We first calculated the distance from each CTCF binding site to the transcriptions start site (TSS) of the nearest downstream gene. Regardless of the evolutionary class or tissue-distribution of the binding site, we observed a large proportion of sites near the TSS (median − 11 kb) (Fig. 4a). The majority of the remaining sites lie more than 100 kb upstream of the TSS, and CTCF binding is depleted directly downstream of the TSS within the gene body.

To further explore the possible functional impact of evolutionarily distinct CTCF binding classes, we examined the patterns of active histone modifications near these regions. Specifically, we mapped their genome-wide co-localisation with H3K4me3 and H3K27ac histone modifications. For all evolutionary classes of CTCF binding sites, 12–13% bind within active promoter sequences as characterised by the presence of the H3K4me3 histone modification, and a further 3–6% co-bind within active enhancers characterised by the presence of the H3K27ac histone modification alone. These co-localisations are higher than observed for a matched set of random genomic regions (binomial test with Bonferroni correction, all *p* values < 0.001) (Additional file 3: Figure S3A). We next investigated whether CTCF binds close to these regulatory elements, which could also indicate a functional role. 75% of all CTCF sites were found within a 50-kb distance of regulatory element (Additional file 3: Figure S3B), with promoters being the nearest regulatory element to 52% of both *musculus*-common and BL6-specific CTCF binding sites and 59% in BL6 tissue-shared sites. We further found that the majority of CTCF binding sites, regardless of evolutionary origin, are located significantly closer to active regulatory elements than a matched set of randomised genomic sequences, with median distances of 22.7 kb and 17.7 kb to their nearest promoter and enhancer, compared to 105.6 kb and 75 kb for random regions, respectively (Mann-Whitney *U* test, *p* value = 0.02) (Fig. 4b). Taken together, the results indicate the potential for evolutionarily young CTCF sites involvement in regulating gene expression through cooperation with existing active *cis*-regulatory elements.

To investigate whether the various evolutionary types of CTCF binding sites are associated with changes in gene expression, we retrieved liver RNA-seq data from Goncalves et al. [52] for both subspecies to identify differentially expressed (DE) genes and their association with CTCF binding. Around 25% (6725) of 1-to-1 orthologous genes were significantly (FDR < 0.05) differentially expressed between the two subspecies (Fig. 4c). We defined a DE gene as associated with CTCF binding if it was within 50 kb of a CTCF binding site. For all DE genes, 91% (6101) were associated with the binding of a CTCF site including 9% (624) overlapping a binding site (Fig. 4d). CTCF binding events associated with DE genes were proportionally similar for all evolutionary types of sites, with 52–58% of all CTCF sites binding in ± 50 kb window compared to only 30% for a matched set of random genomic regions (chi-square tests with Bonferroni multiple testing correction, all *p* values < 2.2e−16) (Additional file 3: Figure S3C). Of the DE genes, 86% (5041) were found to be associated with a *musculus*-common CTCF binding site and 14% (822) with a BL6-specific site, significantly more than would be expected given their overall genomic distribution (chi-square test, *p* value = 4.04e−16) (Additional file 3: Figure S3D).

To further investigate the potential differing effects of CTCF binding on the up- and down-regulation of genes, we investigated their association to different active regulatory regions. DE gene start sites overlapped histone modifications characteristic of active promoters 85.5% (5188) of the time and 11,652 enhancers were within ± 50 kb from DE genes associated with CTCF occupancy. To analyse the effect of these various regulatory regions, we divided the DE genes based on their regulatory profile (Fig. 4e): 43.3% of DE genes overlapped an active promoter signal, had at least one CTCF binding event and had an active enhancer signal within 50 kb (CTCF + promoter and enhancer); 35.5% DE genes had an active promoter and a CTCF binding site (CTCF + promoter), 10.4% including CTCF with a nearby enhancer and 10.8% with only a CTCF binding site. When stratified by the evolutionary origin, DE genes associated with BL6-specific sites appear to be more downregulated across all regulatory classes except for CTCF + enhancers, albeit this difference was only significant for DE genes with a CTCF + promoter (chi-square tests with Bonferroni multiple testing correction, *p* values < 0.001). On the other hand, *musculus*-common sites are more upregulated, reflecting the overall general trend for all CTCF-associated DE genes across all regulatory classes (Fig. 4e). Upregulated DE genes showed no statistically significant gene enrichments, but downregulated genes with active promoters did (Additional file 3: Figure S3E). The top biological process associated with all downregulated genes was macromolecule metabolic process (GO:0043170), which was also found to be significantly represented within all DE genes with either an associated CTCF and promoter or CTCF, promoter and enhancer (Additional file 3: Figure S3E), as well as downregulated DE genes with BL6-specific binding and active promoter. These results suggest that all evolutionary classes of CTCF binding sites equally bind in the vicinity of other *cis*-regulatory elements and might play similar roles in downregulating gene expression.

Taken together, these observations illustrate that recently evolved, subspecies-specific CTCF binding sites mirror the pattern of *musculus*-common sites in their distribution around genomic features and may perform similar functions regardless of tissue-specificity.

### Recent BL6 tissue-shared CTCF binding is TAD-boundary associated

In order to establish the orientation of CTCF binding at topologically associating domains (TADs), we scanned *musculus*-common, BL6-specific tissue-shared and tissue-specific CTCF sites for the presence of the M1 canonical binding motif [35]. Whereas 95% of all *musculus*-common sites harbour an instance of the motif within their sequence, significantly fewer BL6 tissue-shared sites (91%) and BL6 tissue-specific sites (85.4%) contain the motif (binomial test, *p* values 7.1e−07 and < 2.2e−16 for tissue-shared and tissue-specific, respectively) (Additional file 4: Figure S4A *left*). Of BL6-specific tissue-shared sites that did not have the canonical motif, 40% harboured one or more alternative motifs (Additional file 4: Figure S4A *right*, obtained from the CTCFBSDB 2.0 database [53]), compared to a third of tissue-specific sites (Additional file 4: Figure S4A *middle*). Furthermore, in BL6 tissue-specific sites the canonical motif was found at increased distances from the summit than in *musculus*-common sites and coincided with a reduction in motif score (Kruskal-Wallis *p* values all < 2.2e−16 for motif score and distance; Mann-Whitney *U* tests, *p* values range from 5.3e−08 to < 2.2e−16) (Additional file 4: Figure S4B). Only CTCF sites that harbour the canonical motif were used to study CTCF association with TAD boundaries.

**Fig. 5:**
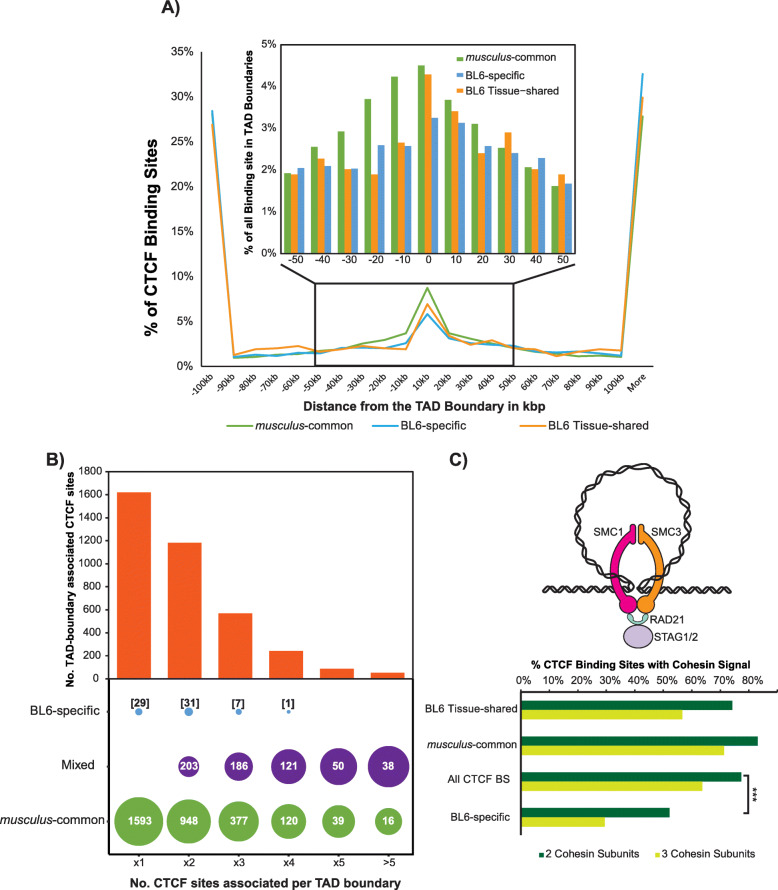
Recent BL6 tissue-shared CTCF binding is TAD-boundary associated and efficiently recruits cohesin. **a** Plot of the distances of CTCF binding sites to the nearest topologically associated domain (TAD) boundary reported in Bonev et al. [54] for each evolutionary type of site. The inner box focuses on the region − 50 kb and + 50 kb from the nearest TAD boundary and shows the percentage of CTCF sites from each evolutionary type at 10 kb intervals. **b** TAD boundaries with increasing number of associated CTCF binding tend to comprise of a cluster of both *musculus*-common and BL6-specific binding sites. The bar plot (*top*) shows the number of CTCF sites associated with each TAD boundary (i.e. within 50 kb) and in a favourable motif orientation from cortical neurons. The circles (*bottom*) display the evolutionary make-up of sites associated with TAD boundaries, with the number of sites per category denoted inside the circles. The size of the circles indicates the proportion of CTCF sites belonging to each of the three classes (BL6-specific only, *musculus*-common only and a mixture of both) at increasing number of TAD boundary-associated CTCF sites. **c** The percentage of all CTCF binding sites and those of different evolutionary classes for which colocalisation with a cohesin complex protein was found. The asterisks indicate the significance of a Chi-square goodness of fit test for 2 cohesin subunits colocalising with CTCF between all CTCF sites and those found to be subspecies-specific (*p* value = 2.8 × 10^−9^). The schematic diagram next to the bars is an overview of the structure of the cohesin complex

We next examined the possible contribution of different evolutionary classes of CTCF to large-scale 3D genome structure. We took advantage of HiC experiments that determined the position of TAD boundaries in the liver [29] and cortical neurons [54] to analyse the distribution of CTCF binding sites in relation to TADs. The distribution of CTCF binding distances to TAD boundaries is similar between all evolutionary categories of binding sites in both tissues (Fig. 5a for cortical neurons and Additional file 4: Figure S4C for liver). Specifically, the majority of both *musculus*-common and species-specific CTCF binding sites were located far (> 100 kb) from TAD boundaries, with slight enrichment in the immediate vicinity of the boundaries. To explore binding at TAD boundaries in more detail, we limited our analysis to ± 50 kb around TAD boundaries, where 30% (> 10,000) of CTCF sites are bound. As expected, the enrichment of *musculus*-common sites at TAD boundaries was greater than for subspecies-specific sites [29, 44]. Though the majority of CTCF binding sites at TAD boundaries are *musculus*-common, subspecies-specific had proportionally similar enrichments at TAD boundaries (Fig. 5a) regardless of their tissue-distribution. Despite the lower number of total TADs identified in the cortical neurons [54] (a 33% reduction in the total number of TADs), the proportion of different evolutionary classes of CTCF binding closely associating with TAD boundaries remained roughly the same (Additional file 4: Figure S4D). This suggests that some TAD boundaries, despite mostly being invariant across tissues [30], may be in part maintained by tissue-specific CTCF binding.

*Musculus*-common CTCF binding sites are significantly overrepresented within a ± 50-kb window of a TAD boundary compared to BL6-specific sites, both tissue-shared or otherwise, based on their overall respective genomic distributions (chi-square test, *p* value < 2.2e−16) (Additional file 4: Figure S4E *bar*). To investigate whether these TAD-boundary-associated CTCF sites could be involved in TAD formation, we identified the canonical CTCF motif orientation for binding sites of all evolutionary types. Of TAD-boundary-associated *musculus*-common CTCF binding sites, 70% were of a favourable orientation (see diagram in Additional file 4: Figure S4E), compared to only 57% of BL6-specific sites (chi-square tests with Bonferroni multiple testing correction, all *p* values < 2.2e−16) (Additional file 4: Figure S4E *pie charts*). As with distance to TAD boundaries and motif scores, a large portion of tissue-shared BL6-specific CTCF binding sites have a favourable orientation at ± 50 kb of the TAD boundary (66%), not significantly different than *musculus*-common CTCF sites (chi-square tests with Bonferroni multiple testing correction, *p* value = 0.21).

Of the TAD boundary-associated sites in a favourable orientation, 33% (1622) are the only favourably oriented site in ± 50 kb window from the boundary and are almost all *musculus*-common (> 98%) (Fig. 5b). However, as the number of favourably oriented TAD-boundary-associated CTCF sites increases, the proportion of *musculus*-common only TAD boundary sites decreases. Although very few BL6-specific sites are the only favourably oriented CTCF site near TAD boundaries, they more commonly appear in clusters of both *musculus*-common and BL6-specific binding sites (Fig. 5b). This extends previous results on more diverged mouse species [29, 44] and indicates that even evolutionarily younger CTCF binding sites cluster with older *musculus*-common binding and may contribute to the maintenance of higher-order genomic architecture [29, 44].

### Recently evolved tissue-shared CTCF binding efficiently recruits cohesin

To form both large-scale and smaller-scale 3D genome structure, CTCF can help stabilise cohesin and form a chromatin loop (Fig. 5c, diagram). To quantify the extent to which subspecies-specific CTCF binding can recruit cohesin, we determined the level of co-location of CTCF and cohesin in BL6 mice. We used our previously published ChIP-seq data from two biological replicates of adult mice livers for three proteins that form the cohesin complex: RAD21, STAG1 and STAG2 [55]. RAD21 is necessary for the formation of the core ring of the cohesin complex, which is completed with either STAG1 or STAG2 [56, 57]. We defined colocalised events as those where at least two cohesin subunits overlap with CTCF binding. All evolutionary classes of CTCF binding sites colocalised with cohesin, with the highest fraction of colocalisation (~ 80%) observed for *musculus*-common CTCF sites. A significantly smaller portion of BL6-specific CTCF sites (~ 50%) colocalised with at least two cohesin subunits (chi-square test *p* value = 2.8 × 10^−9^). However, the tissue-shared subset of the BL6-specific sites (i.e. the ones bound in all five tissues in Fig. 3e) colocalised with cohesin at the same level as the set of all CTCF binding sites, and only slightly less than that of the *musculus*-common sites (Fig. 5c). The increased ability of BL6-specific tissue-shared binding sites to recruit cohesin in comparison to their more tissue-restricted counterparts suggest that there is a fundamental difference in the function of these recently evolved CTCF binding sites.

We next investigated whether the differences in cohesin recruitment in BL6 tissue-specific sites can be explained by lower overall CTCF ChIP signal enrichment. At the top 10% of the signal, almost all CTCF sites are associated with cohesin recruitment to the DNA, regardless of the evolutionary or tissue-specificity of the binding site (Additional file 4: Figure S4F). At the bottom 10% tier of the signal, only 56% of *musculus*-common CTCF sites are associated with cohesin, and even fewer at BL6 tissue-specific sites (38%). This indicates that reduced cohesin recruitment at lower ChIP signal is more pronounced in BL6 tissue-specific sites. The reduction of cohesin recruitment is generally increased even at low CTCF ChIP signal values for BL6 tissue-shared sites. This may in part due to the small number of CTCF sites at lower tiers of signal intensity, though the proportion of BL6-specific tissue-shared sites is similar across all signal intensities (Additional file 4: Figure S4G). When compared to all BL6-specific sites, those that are tissue-shared make up over half of the sites in the top 40% range of the signal, but the tissue-specific sites become more predominant in the lowest 50%. These results show that, though there is an overall difference in the ChIP-seq intensities across different evolutionary classes of CTCF binding sites, the correlation between CTCF ChIP-seq intensity and cohesin scales similarly within each class.

### CTCF contributes to a lineage-specific interferon gene and regulatory expansion

**Fig. 6:**
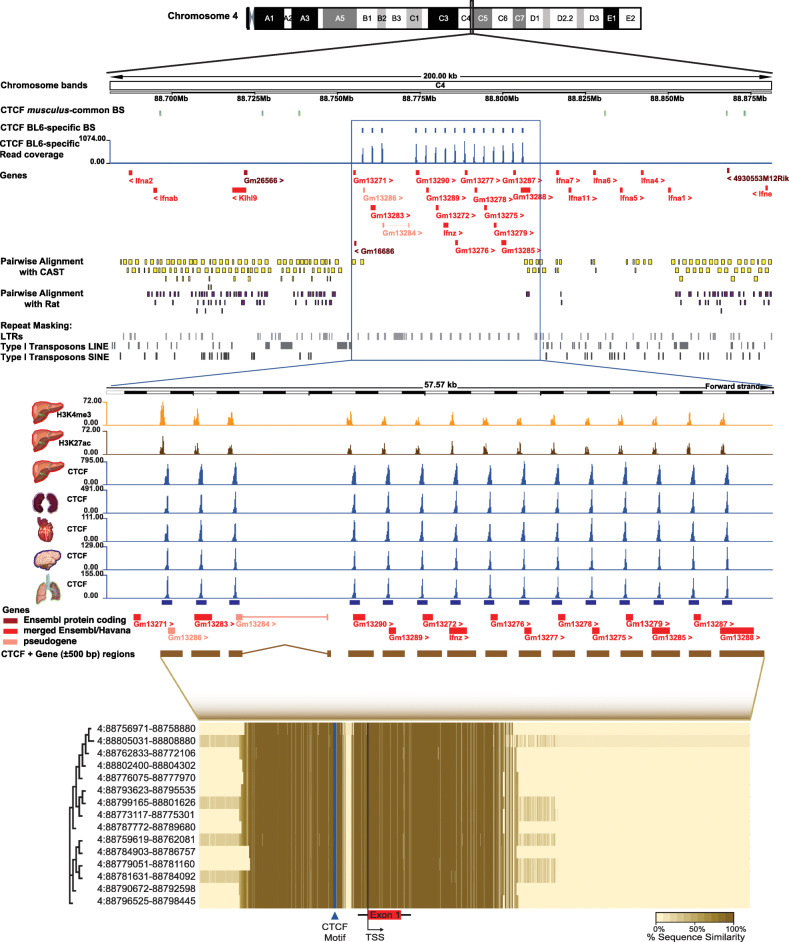
Evidence of a tandem duplication event of BL6-specific CTCF binding sites on Chromosome 4 in multiple tissues linked to the expansion of a family of interferon genes. **a** A summary view of 200 kb on Chromosome 4 band C4 of the BL6 genome. The top two tracks show the CTCF-cohesin bound genomic regions in *musculus*-common and BL6-specific tissue-shared sites. The next track in blue shows the read coverage for CTCF BL6-specific tissue-shared binding sites. All genes in the 200-kb window are denoted below in red, with arrowheads indicating the direction of transcription. The pairwise alignments of the BL6 region to CAST (yellow) and rat (purple) show a noticeable lack of any orthologous regions in either species. The repeat content of the genomic region is shown in the bottom three grey tracks, illustrating the noticeable lack of any large-scale repeat elements in the highlighted region. **b** A zoomed-in view of the 57.6 kb region 4:88752534-88810107 in which BL6-specific CTCF-cohesin colocalised binding was observed. The top two tracks in orange and brown indicate read coverage signal from H3K4me3 and H3K27ac, respectively. The next five tracks in blue indicate CTCF ChIP-seq read coverage in five tissues (described in Fig. 2) and illustrated that these CTCF sites are also bound in other tissues. The 15 interferon zeta cluster genes are shown below the tracks. **c** Heatmap of the extent of sequence between similarity in a multiple sequence alignment of the 15 CTCF-cohesin binding sites and interferon zeta genes on the C4 band of BL6 chromosome 4. The dendrogram on the left is generated from the overall sequence similarity of each region. Coordinates represent the start and end positions of each binding site

We next examined the genomic positions of CTCF binding sites colocalised with cohesin and noticed an extreme but interesting example. Chromosome 4 of the BL6 genome contains a cluster of 15 CTCF-cohesin sites colocalised and tissue-shared binding sites within 58 kb, with all sites of similar lengths and with comparable ChIP-seq signal strength (Fig. 6a). There is no detectable presence of either *musculus*-common or tissue-specific CTCF binding, indicating that this region is uniquely bound in a subspecies-specific and tissue-shared manner. This genomic cluster is unlikely to be the result of a genome assembly artefact as it is contained within a single clone within the reference BL6 mouse genome assembly (https://www.ebi.ac.uk/ena/data/view/AL928605). Interestingly, each of the 15 CTCF-cohesin sites within this region is upstream from a transcription start site of an immunity-related gene of the type 1 interferon zeta (Ifnζ) family. We have manually re-evaluated the annotation for all of the genes in the cluster and found the annotation to be largely correct (see the “Methods” section). The majority are novel/predicted protein coding genes are part of the comprehensive GENCODE annotation [58], with supporting transcript level evidence [59, 60]. The 15 Ifnζ genes are also known as limitin and have previously been identified as a mouse-specific gene family expansion [61, 62].

To more closely investigate the evolutionary origin of this gene cluster, we examined whole genome alignments within the rodent clade. Most of the BL6 gene cluster has no alignable regions in the genome of CAST, rat (Fig. 6a) or any of the other 13 mouse strains/species in pairwise alignments available in Ensembl version 91 [63]. This cluster was characterised by a strikingly different distribution of transposable elements compared to the neighbouring regions. There was no detectable SINE or Long Interspersed Nuclear Elements (LINE) transposons, with the only repeat elements present all belonging to the Long Terminal Repeat (LTR) ERVK subfamily (Fig. 6a). These LTR-ERVKs were between 450 and 550 bp in length and located in intergenic regions, around 500 bp up/downstream from gene bodies. The only detectable repeats in this region were six short simple repeats (average length 50 bp), which are unlikely to be from transposable elements. The LTR-ERVK elements did not colocalise with binding of the CTCF-cohesin complex or CTCF alone. The LTR-ERVKs, CTCF-cohesin bound regions and genes in this cluster exhibit high sequence similarity for large portions of their lengths (Fig. 6c). Given that LTRs have been shown to have regulatory activity in gene family expansions [64], we examined the enrichment of specific histone modifications from ENCODE [48]. Each CTCF-cohesin binding site was also found to overlap in the liver with H3K27ac predictive of regulatory activity [65] and H3K4me3 predictive of promoter function [66] (Fig. 6b). Hi-C maps of 3D genomic interactions of the 200-kb locus [54, 67] show that these genes belong to the same TAD, with only weak long-range interactions to neighbouring genomic regions (Additional file 5: Figure S5 *top*), suggesting that they are co-regulated. Examination of RNA-seq data for the thymus, spleen and liver available in Ensembl show low levels of transcriptional activity for genes in this cluster compared to neighbouring genes from outside the cluster (Additional file 5: Figure S5 *bottom*). These results show that the Ifnζ gene cluster is not only *musculus*-specific, but a more recent subspecies-specific tandem duplication event restricted to BL6. The gene cluster expansion and/or regulation of gene expression might have been facilitated by the presence of recently evolved transposable elements.

Interestingly, despite the lack of alignable regions in more closely related species, LASTz whole genome alignments with other eutherian mammals revealed that 14 of the 15 genes in the BL6-specific cluster are aligned with high coverage (50–100%) to a single gene in the pig [63, 68]. The predicted pig protein-coding gene is located on chromosome 1 (ENSSSCG00000039987) and has 13 paralogues within a 470-kb cluster, albeit separated by 24 intervening genes (Additional file 6: Figure S6). All the genes belong to the Ensembl protein family PTHR11691 (Interferon Precursor), which has only a single member outside the cluster. Similar to the BL6 cluster, this region is enriched with LTRs punctuating the intergenic regions. Compared to the surrounding genomic regions, this pig cluster has lower GERP conservation scores with less constrained elements, indicating a more recent evolutionary origin. Motif discovery analysis of 1 kb regions around the 14 gene paralogues revealed that almost all have a CTCF binding motif less than 100 bp from the transcription start site (Additional file 6: Figure S6C). These results support previous suggestions of lineage-specific expansions of IFNδ in the porcine lineage and IFNζ in the mouse lineage from a more ancient IFN gene [62] and may be an example of convergent evolution.

## Discussion

Recent studies have demonstrated the regulatory potential of species-specific transposable element insertion in primates, especially in tissue-specific contexts [49, 50]. In particular, the emergence of novel CTCF binding by repeat expansion is a mechanism known to have repeatedly reshaped the genomes of highly diverged mammals [32, 35]. In the mouse genome, very recent waves of expansions of SINE B1 and SINE B2 transposable element subfamilies are known to have created many novel CTCF binding sites not present in rat [37, 69]. Here, we use two very closely related mouse subspecies, *Mus musculus domesticus* (BL6) and *Mus musculus casteanus* (CAST), separated by only 1 million years of evolution to reveal the speed of repeat expansion associated CTCF binding and to suggest potential functions for these young sites.

It has previously been shown that a large fraction of hominidae-specific transcription factor binding sites, when compared to ancestral human-mouse shared ones, are enriched near genes implicated in specific pathways and may therefore have distinct biological functions [70]. However, mouse and rat species-specific CTCF sites have comparable functional effects to shared sites on chromatin domain demarcation and transcriptional regulation [35]. To investigate the potential biological function of even younger transcription factor binding sites, we compared the genomic locations and gene function enrichment of subspecies-specific CTCF sites with sites common between BL6 and CAST. We found that they are mostly indistinguishable in the gene features they bind, distance to transcription start sites or TAD boundaries. Our results illustrate how these evolutionarily young CTCF sites have been captured into operational regions of the genome and adopted functions similar to *musculus*-common sites. This observation is in line with the observation that species-specific CTCF binding sites cluster with species-shared sites to provide functional redundancy [44]. The final conclusions on the biological function of lineage-specific CTCF sites will require more targeted in vivo studies, such as CRISPR-Cas9 of individual CTCF sites or conditional knockdowns of CTCF.

It has also been shown that tissue-shared CTCF binding is more conserved than tissue-specific CTCF binding [70, 71], but the functional differences between tissue-shared and tissue-specific young CTCF sites have not yet been investigated. By determining the tissue-distribution of CTCF binding of evolutionarily young CTCF sites, we found significant differences between tissue-shared and tissue-restricted sites. Most subspecies-specific sites are restricted in their tissue distribution, but many are still bound across multiple tissues originating from all three germ layers. These subspecies-specific tissue-shared sites are almost as likely to be colocalised with cohesin as *musculus*-common, and far more than other subspecies-specific sites. This suggests that these tissue-shared, subspecies-specific sites have a greater regulatory potential and are more likely to adopt functional signature than their cell-type-specific counterparts. Due to the resolution of available Hi-C experiments for determining 3D genome structure, we could not establish whether pairs of colocalised CTCF-cohesin subspecies-specific sites are implicated in forming chromatin loops. Most pairs of sites were too close to *musculus*-common CTCF-cohesin regions, or too close to each other, to be able to establish their contacts. Further chromatin capture experiments with higher resolution are needed to investigate loops associated with these sites and to establish the extent to which CTCF subspecies-specific sites contribute to transcriptional regulation either in tissue-shared or tissue-restricted scenarios.

There have been previous reports of clustered expansion of functional genes of the interferon alpha family between the BL6 and 129/5v mouse strains [72]. Similarly, the expansion of the *Abp* gene cluster is well described in mice and is associated with transposable elements [37, 64]. We found an example of a BL6-specific gene cluster and CTCF binding expansion of the type 1 interferon zeta family is associated with a specific LTR expansion, and evidence of a similar expansion in pig. The LTRs may either have served to duplicate the genes through non-allelic homologous recombination or helped provide binding sites for regulatory factors [73]. Within this gene cluster, subspecies-specific CTCF binding colocalised with cohesin and histone modifications indicative of active promoter function, and the genes show transcriptional activity. This suggests that CTCF may have helped established 3D genome structure and transcriptional regulation within the locus. The mouse gene cluster has not been well-described in the literature, though it is known to be a mouse-specific expansion [62]. Characterisation of the larger area around the subspecies-specific gene cluster revealed that this interferon locus in the mouse has a number of orthologues in the human interferon locus on chromosome 9, but the smaller subspecies-specific cluster was completely overlooked in part due to concerns of an assembly artefact [72, 74]. Our detailed manual curation of the genes within the cluster, and confirmation that the region is contained within a single BAC clone, disprove assembly artefacts in the region. Further exploration of the genes in the locus is difficult due to many subsequent changes to names or gene IDs, and due to changes to the protein-coding status of some genes as transcriptional evidence improved. The evolution of species-specific regulatory elements along with lineage-specific gene clusters, as we show in this paper, may yet turn out to be a more common than thought.

## Methods

Liver ChIP-seq libraries from two closely related *Mus* subspecies were obtained for *Mus musculus domesticus* (C57BL/6 J *or* BL6) from Thybert et al. [37], and for *Mus musculus castaneus* (CAST) from Kentepozidou et al. [44], each with three biological replicates. Liver ChIP-seq libraries for CEBPA and FOXA1 were retrieved from Stefflova et al. [47] for both subspecies in biological triplicate. Liver ChIP-seq libraries for H3K4me3 and H3K27ac were obtained for C57BL/6 J (BL6) from Wong et al. [75], each with three biological replicates. Liver RNA-seq libraries for six biological replicates for both subspecies C57BL/6 J and *M. m. castaneus* were retrieved from Goncalves et al. 2012 [52]. ChIP-seq data for three cohesin-complex subunits (Rad21, STAG1 and STAG2) in the liver, two biological replicates for each subunit, from adult male mice and matched controls were retrieved for BL6 from Faure et al. [55]. TAD boundary domains were retrieved from Vietri Rudan et al. [29] and Bonev et al. [54]. CTCF and histone modifications H3K4me3 and H3K27ac ChIP-seq libraries across adult mouse tissues were retrieved from the ENCODE Project data repository for BL6 adult (8 weeks) male mice and 13 tissues: liver, lung, bone marrow, bone marrow macrophages, cortical plate, cerebellum, heart, kidney, thymus, spleen, olfactory bulb, small intestine and testis [48].

### ChIP-seq sequence alignment and peak calling

All libraries were retrieved as raw ChIP-seq FASTQ reads were subject to quality control using standard parameters in FastQC version 0.11.5 [76]. Good quality reads (min Phred score ≥ 30) were subsequently aligned to most recently available genome assembly in Ensembl (GRCm38 for BL6 and CAST_EiJ de novo assembly downloaded from ftp://ftp-mouse.sanger.ac.uk/, and available in Ensembl version 84 for CAST). We aligned the sequence reads to the reference genomes using BWA version 0.7.12 [77] for each biological replicate and control. Aligned reads were afterwards filtered for duplicate and non-unique reads, sorted and indexed using SAMtools version 1.2 [78]. CTCF binding sites were identified by peak calling from aligned sequence reads using MACS version 2.1.0 [79] with a *p* value threshold of 0.001 to call peaks representing CTCF bound regions. Peaks found in at least two biological replicates were used for downstream analysis. Motif analysis focused on the summit point (± 50 bp) of each identified CTCF binding sites using the MEME suite version 4.10.2 [80, 81]. The most overrepresented motif found in each dataset is reported in Fig. 1b. CTCF binding site characterisation in terms of gene feature occupancy and proximity to downstream gene bodies was performed using the annotatePeaks.pl tool from HOMER (Hypergeometric Optimization of Motif EnRichment) suite (v4.11) with annotation from the most recent mouse genome assembly (GRCm38) [82].

### Interspecies comparisons

Interspecies comparison between BL6 and CAST was performed first using a multiple alignment of 15 de novo assemblies of laboratory and wild-derived strains genomes within *Mus musculus* [42, 46]. Orthologous regions with a CTCF binding site present in orthologues alignment regions in both species was considered a “*musculus*-common” site, while sites found in only one of the species, but absent from the other, was considered “subspecies-specific”.

### Repeat masking of CTCF binding sites

CTCF binding regions from *musculus*-common and species-specific sets of the data for both species were screened for repeat elements using RepeatMasker 4.0.5 [83] using the rodent repeat libraries from RepBase (v20140131) for the two murine species, with the cross_match search engine, masking for interspersed and simple repeats. Fragmented hits found to be part of the same repeat were merged as one.

To calculate the background representation of the 4 superfamilies of transposable elements (TEs) (SINEs, LINEs, LTRs, DNA transposons) in the mouse genome for comparison with *musculus*-common and BL6 subspecies-specific sites enrichment, the sum total of the sequences occupied by each TE superfamily divided by the total length of the genome. We retrieved the full set of TEs for the C57BL/6 J mouse genome from those published in Thybert et al [37]. To derive the random set of genomic sequences, we used the BEDTools version 2.2.5.0 [84, 85] shuffle tool to generate sequences equal in number and length to the total number of CTCF peaks obtained from our ChIP-seq libraries. Random sequences were matched for the chromosomes but were non-overlapping with any of the genomic regions in the CTCF peaks set.

We used the intersection between CTCF peaks and the full set of the four TE superfamilies to derive the proportion of sequence occupied in each CTCF binding site and the relative age of the repeat element present. To determine the fraction of sequence occupied, we used BEDTools intersect 2.2.5.0 with the option -wo to return the overlap between the peak sequence and the repeat, then divided the overlap by the total length of the peak to obtain the percentage of sequence occupied by TE for every individual peak and random sequence. We estimated the relative age of a repeat element from the percentage of sequence substitutions in each repeat from the consensus sequence of that element. The higher the percentage of substitutions in TEs compared to the consensus, the older the sequence is.

### Repeat content analysis of liver-specific transcription factors (TF) binding sites

Raw ChIP-seq libraries from Stefflova et al. [47] were used for repeat content comparison to other two liver-specific transcription factors, CEBPA and FOXA1 for both mice subspecies. In each case, three biological replicates from different 8-week-old male mice and a matched control liver sample from another animal. Peaks found in at least two biological replicates out of the three were used for all downstream analysis. The raw FASTQ sequence was run through the same pipeline outlined earlier for CTCF peak calling. Interspecies comparison and repeat masking were also performed as described above for CTCF.

For studying the correlation between repeat content and the signal intensity of the TF binding site, all datasets for each transcription factor and CTCF in both subspecies were subsequently divided into ten 10% bins based on descending intensity of the ChIP-seq signal for each of the three evolutionary classifications: *musculus*-common, BL6-specific and CAST-specific. The repeat content for each bin of TF binding sites was then determined using the methodology detailed above.

### Cross-tissue analysis of species-specific CTCF binding

CTCF ChIP-seq data for BL6 adult (8 weeks) male mice were retrieved from the ENCODE Project data repository [48] for 12 tissues: lung, bone marrow, bone marrow macrophages, cortical plate, cerebellum, heart, kidney, thymus, spleen, olfactory bulb, small intestine and testis. We additionally used ENCODE libraries for the liver as a technical replicate to identify CTCF binding sites common in multiple tissues. In each case, two biological replicates from different 8-week-old male mice were used, and a matched control tissue sample from another animal. Peaks found in both of the two biological replicates were used for downstream analysis. The raw FASTQ sequence data were used and run through the same pipeline as outlined earlier for liver CTCF peak calling.

We used the overlap between our liver-derived CTCF peaks and the peaks from every ENCODE tissue to determine the tissue-sharedness of *musculus*-common and BL6-specific binding sites, using BEDTools intersect 2.2.5.0 with the options -wa -wb. UpSet plots were generated using the ComplexHeatmap package in R [86].

To calculate the CTCF diversity index across ENCODE tissues, we used the log10 of the *p* value at peak summit computed by MACS version 2.1.0 during the peak calling step. For each liver-derived CTCF peak, if that peak was bound in an ENCODE tissue, the *p* value for the binding of CTCF was retrieved. These values were subsequently used to calculate the Shannon Diversity Index for each tissue using Vegan package in R [87]. CTCF occupancy conservation across tissues was calculated as the fraction of CTCF peaks whose occupancy is conserved within each bin of the Shannon diversity index.

Based on the results of the ENCODE tissue analysis, BL6-specific sites were then defined as tissue-shared or tissue-specific. Tissue-shared sites were CTCF binding sites found to be the intersection of all BL6-specific binding sites from the top four ranking tissue, plus the ENCODE liver technical replicate. All other BL6-specific sites were deemed tissue-specific.

### CTCF occupancy at TSS and proximal active regulatory elements

CTCF binding sites regions were analysed with GREAT version 3.0 [88] using default parameters to determine the distance from each CTCF site of each category to the nearest transcription start site (TSS). All CTCF sites more than ± 100 kb from the nearest TSS were pooled together.

Liver ChIP-seq libraries for H3K4me3 (a histone modification predictive of active promoter regions) and H3K27ac (a histone modification predictive of active promoters and enhancers [65]) were obtained for C57BL/6 J (BL6) from Wong et al. [75], each with three biological replicates. Reads were aligned, filtered and peaks were called using the methodology explained above for liver CTCF binding sites. Only peaks common to a minimum of two replicates were used to define active regulatory elements. A promoter region was defined by the localisation of either H3K4me3 only, or with overlapping H3K27ac signal, whereas enhancers were defined by the presence of the histone modification H3K27ac alone within the peak region.

Co-localisation of TF binding sites with regulatory elements was defined using an intersection of at least 1 bp between the TF binding site and the regulatory element. BEDTools intersect 2.2.5.0 with the option -wa -wb was used to retrieve all overlaps between binding sites and active regulatory regions.

CTCF proximity to active regulatory regions was measured using BEDTools closest 2.2.5.0, with the options -D ref. and -mdb all against all active regulatory region to return only the closest enhancer/promoter but not both at the same time. We excluded any sites whose distance to the CTCF binding site is 0 (i.e. overlaps the binding site) as these sites have already been considered for the co-localisation analysis outlined earlier.

### RNA-seq and differential expression analysis

RNA-seq libraries for all biological replicates for BL6 and CAST were aligned to their respective genome using STAR 1.5.2 [89] with default parameters. HTSeq [90] was used to count raw RNA-seq reads mapping back to annotated genes for both subspecies. Raw reads for all 12 biological replicates were passed to DESeq2 1.28.1 [91] for differential gene expression analysis to calculate fold change between CAST and BL6, using ‘apeglm’ for Log Fold Change (LFC) shrinkage. Genes were deemed differentially expressed (DE) using adjusted *p* value cut off and false discovery rate (FDR) of 0.05.

The distance from each CTCF binding site to its nearest DE gene was calculated using the BEDTools closest 2.2.5.0 tool. Definitions of up/downregulation were based on the log2 fold change of expression in BL6. If more than one CTCF binding site was found in a 50-kb window of the gene body, their evolutionary class-assignment was performed as follows: if there were more *musculus*-common sites than either of the BL6-specific varieties, the DE gene was considered associated with a *musculus*-common CTCF site. If no *musculus*-common CTCF site was present (or in few cases, in equal number to either of the two types of BL6-specific sites), the DE gene was said to be associated with BL6-specific site, tissue-shared or otherwise based on the type of site(s) present.

To identify active regulatory elements nearby CTCF-associated DE genes, we used the definitions of active promoters and enhancers set above in section (CTCF occupancy at TSS and proximal active regulatory elements). An active promoter was assigned to a DE gene if the promoter sequence overlapped the gene start. In all cases, only a single active promoter sequence was assigned to each DE gene when identified. Owing to their orientation-free mode of action, there were no restraints on enhancer assignment in terms of position or number in the ± 50-kb window.

Gene enrichment analysis for all sets of DE genes was performed using the analysis tool from the PANTHER Classification System [92–94]. Gene Ontology (GO) search for most over/underrepresented genes in each set at an FDR cut-off value of 0.05 using Fisher’s exact test.

### TAD boundary association analysis

FASTA sequences from the CTCF binding sites were obtained using BEDTools getfasta 2.2.5.0. Sequences were then scanned for CTCF canonical binding motif (M1) JASPAR database (JASPAR motif MA0139.1) using the MEME suite motif scanning function Find Individual Motif Occurrences (FIMO) with default parameters [80, 81]. We used FIMO-assigned CTCF motif orientation and motif scores for further downstream analysis. In the case of CTCF binding sites with more than one instance of the M1 motif in their sequences, the motif that is closest to the summit of the replicate where the peak signal was at its highest, as defined by the output of peak-calling step using MACS, was selected for downstream analysis. CTCF sites with 0 motif instance in the BL6-specific set of CTCF sites were subject to further motif scanning using alternative CTCF motifs, retrieved from CTCFBSDB 2.0 [53] (http://insulatordb.uthsc.edu/download/CTCFBSDB_PWM.mat). Visualisation of both the canonical motif and the alternative motifs was performed using the position weight matrices for each motif as obtained from their respective sources and carried out using the PWMScan from PWMTools [95].

To calculate the distance from each CTCF binding site to the nearest up/downstream TAD boundary, mouse liver and cortical neurons TAD boundary data from Vietri Rudan et al. [29] and Bonev et al. [54], respectively, were used. For the liver data, we defined the TAD boundary as the start or end nucleotides for every TAD in that dataset. The distance from each TF binding site and its nearest TAD boundary was measured using the BEDTools closest 2.2.5.0 tool.

### CTCF-cohesin colocalisation analysis

Genomic regions where at least two cohesin subunits peaks overlap were merged using BEDOPS version 2.4.30 [96], and cohesin merged regions overlapping with *musculus*-common/BL6-specific/BL6-tissue-shared from our CTCF liver binding sites were identified. The intersection analysis was done for CTCF co-occupancy with two and three subunits, owing to the significantly fewer number of ChIP-seq peaks retrieved from the STAG1 data.

To investigate the correlation between the evolutionary/tissue-specificity type and cohesin-recruitment by CTCF, we divided each set of CTCF binding sites into ten 10% bins based on descending ChIP-seq signal. ChIP signal in this context referred to the read pileup per peak from the replicate where the peak signal was at its highest, for each of the three evolutionary/tissue-specific classifications: *musculus*-common/BL6-specific/BL6-tissue-shared. CTCF ChIP-seq signal intensity was then compared to the level of cohesin recruitment, defined as the fraction of CTCF sites belonging to each evolutionary/tissue-specific type that falls within a 2-subunit cohesin-bound region.

### Chromosome 4 interferon-zeta gene-cluster analysis

CTCF binding sites coordinates in *bed* format along with ChIP-seq coverage reads in those regions were uploaded for display on the Ensembl genome browser version 89 [63]. These included reads from the liver and the other four tissues, plus ChIP coverage reads from two histone marks for liver, H3K4ac27 and H3K4me3, from the ENCODE data repository. Ensembl genome browser was used to display gene annotations, pairwise alignments with CAST and Rat, repeat elements enrichments for transposons and LTRs and genomic annotations. Sequence similarity for the 15-gene cluster, upstream CTCF binding regions, and the complete 15 constructs of CTCF binding sites plus the gene sequence plus ± 500 bp were determined using Clustal Omega [97], using default parameters.

We utilised the Comparative Genomics tool of the Ensembl Genome Browser to look at the BLASTz/LASTz whole genome alignment between the Chromosome 4 Interferon-zeta gene-cluster and all available pairwise alignments with other organisms [63]. An orthologous gene was found in the pig genome whose target sequence matched 14/15 from the mouse cluster with Query %id of > 50%. We used BLAST/BLAT to scan the pig genome for paralogues to the gene based on sequence similarity. As with the mouse cluster, Ensembl genome browser was used to display gene annotations, repeat elements enrichments for transposons and LTRs and GERP scores. Next, we scanned the 1-kb sequences upstream of each gene’s TSS for the enrichment of motifs using MEME (4.12.0), setting 5 as a maximum number of motifs, and a motif width between 6 and 50 bp. The top 5 motifs from all upstream sequences were subsequently submitted to TOMTOM (2.14.0) to search available databases for annotated motifs to match [80, 81].

The manual annotation review of the locus on chromosome 4 determined that the annotation of the region was essentially correct with only a couple of minor issues identified and corrected. Specifically, Gm16686 was identified as a spurious protein-coding gene that had already been removed from RefSeq and was flagged for removal in GENCODE release M19. RP23-400P11.4 was added as novel interferon pseudogene located at BL6 chr4:88754471-88754678 due to the clear pseudogenic characteristic of a significantly truncated 3′ end. Finally, we reviewed Gm13286, which is annotated as a pseudogene in GENCODE, but considered protein-coding by RefSeq. It has a premature STOP compared to other family members, though it only loses the last 3aa of the typical protein. Based on the GENCODE annotation guidelines, Gm13286 is correctly annotated as a pseudogene although the coding status should be further investigated to make a definitive determination.

## Supplementary information

**Additional file 1: Figure S1.** Repeat content analysis of CTCF binding sites. **A)** Horizontal bar chart shows the proportion of major TE superfamilies in CTCF binding sites that are masked by repeat elements. The top bar represents the percentage each TE superfamily occupies in all repeat masked sequences in the BL6 mouse genome as a background. **B)** 100% stacked bar plot of the proportions of the most common families of SINE TEs in all sequences masked by SINE elements in the different types of CTCF binding sites (The Alu family in the panel refers to the Alu/B1 rodent-specific family). **C)** Box plot of the percentage of sequence mismatches/substitution from the TE consensus sequence within TE superfamilies and across different evolutionary categories of CTCF sites, compared to a matched random set. CTCF BL6-specific sites have significantly lower levels of sequence mismatches in their TE-derived sequences (median = 17%) than either *musculus*-common sites (22%) or randomised genomic regions (21%) (Mann Whitney U test, both *p*-values < 2.2e-16). **D)** The characterisation of the different categories of repeat elements in the binding sites of CTCF and the two TFs from Fig. 2c shows that the CTCF has a distinctive TE profile. While the types of TE in the binding sites of CEBPA and FOXA1 widely vary between them and within their binding sites depending on their evolutionary status, SINE B2-B4 elements almost exclusively make up all TE-derived occupancy in CTCF regardless of subspecies-specificity. The sizes in each plot are proportional to the sequence occupied by each type of repeat element, and their overall proportion of the total binding site sequence masked by TEs according to their evolutionary status.

**Additional file 2: Figure S2.** Tissue distribution analysis of CTCF binding sites. **A)** UpSet plot of the liver-derived CTCF binding sites found across the 12 mouse ENCODE tissues for all sites. The number of sites bound at each combination of tissues is indicated on the y-axis on the top bar chart. The original plot was reduced to these 26 combinations representing only highly tissue-shared and tissue-specific. The rightmost bar on each UpSet plot (*boxed*) indicates the number of CTCF binding sites that were not found to be bound in any other ENCODE tissue library. **B)** Bar plot of the proportion of CTCF binding sites bound in ascending number of tissues in conserved versus BL6-specific sites. The y axis represents the cumulative percentage of binding sites found at the minimum number of tissues on the x axis. The dashed grey line denotes the minimum number of tissues at which 50% sites are shared.

**Additional file 3: Figure S3.** BL6-specific binding exhibits similar regulatory signatures to *musculus*-common CTCF binding. **A)** Bar chart displaying the proportion of CTCF sites in terms of their evolutionary/tissue-specificity at active promoters (H3K4me3 or H3K4me3 + H3K27ac) and enhancers (H3K27ac only) against a matched set of random, non-overlapping genomic regions. **B)** Empirical cumulative density function plot for the distance between CTCF binding sites and their nearest, non-overlapping regulatory element, separated based on their evolution/cell-type specificity. The horizontal dashed grey line indicates the fraction at which 75% of all CTCF sites are at in relation to their distance to the nearest regulatory element, with the vertical marking that distance to 50 kb. The purple line indicates the distance from the random set of regions to their nearest non-overlapping regulatory element. **C)** Plot of the distances of CTCF binding sites to the nearest DE gene against a matched set of random, non-overlapping genomic regions. The inner box focuses on the region − 50 kb and + 50 kb from the nearest DE gene and shows the percentage of CTCF sites from each evolutionary type at 10 kb intervals. **D)** The bar plot (*top*) shows the number of CTCF sites associated (in ±50 kb window) with DE genes for each evolutionary type of site. The circles (*bottom*) break down CTCF-associated DE genes based on their differential regulation (up/downregulation), with the number of sites per category denoted inside the circles. The size of the circles indicates the proportion of CTCF sites belonging to the up/down categories for each evolutionary type of CTCF sites. **E)** Gene enrichment analysis results for the downregulated DE genes associated with BL6-specific CTCF binding and an active promoter (within ±50 kb window of the gene). Gene Ontology (GO) biological processes in *italic* were identified in gene enrichment analysis of all DE genes associated with BL6-specific CTCF binding, an active promoter. The one in **bold** was additionally identified in the analysis of all DE genes associated with BL6-specific CTCF binding, an active promoter and enhancers.

**Additional file 4: Figure S4.** BL6-specific CTCF binding motif characteristics, TAD-boundary occupancy, and cohesin recruitment. **A)** Bar plot (*left*) of the proportions of *musculus*-common and BL6-specific (tissue-shared and tissue-specific) CTCF binding sites lacking the canonical M1 motif in their peak sequence. The asterisks indicate the significance of Chi-square test (*p*-value < 0.0001) for BL6 Tissue-shared sites versus the other two categories. The smaller bar plot (*middle*) shows the proportion of those 0-motif BL6-specific CTCF sites (*boxed*) in which one of the other CTCF motifs from CTCFBSDB 2.0 (*rightmost*) were alternatively identified. **B)** Box plots of the distribution of non-overlapping motifs (> 0) distance to the peak summit in bp (*left*) and the motif scores as calculated from the information content (PWM) of the canonical motif (*right*) according to their evolutionary/tissue-specificity status. **C)** Plot of the distances of CTCF binding sites to the nearest topologically associated domain (TAD) boundary reported in Vietri-Rudan et al. [29] for each evolutionary type of site. **D)** Despite a 33% reduction in the number of TADs between liver (Vietri-Rudan et al. [29]) and cortical neurons (Bonev et al. [54]), the proportion of TAD boundary associated CTCF binding remain concordant in all evolutionary types of binding sites. **E)** The horizontal bar displays the percentage of CTCF sites in ±50 kb window from the nearest TAD boundary for each evolutionary type of site. The pie charts shows the numbers and proportions (*numbers inside*) of CTCF sites with canonical motifs in either “favourable” or “reverse” orientation (see diagram *right*) for each evolutionary type. **F)** Line plot of CTCF-cohesin co-occupancy at matched tiers of signal intensity (reads/site) for CTCF binding sites. All categories of CTCF where within a distinct range of signal for each bin, even though their numbers were variable. Co-occupancy was calculated as the number of CTCF sites that co-localise with a 2-subunit cohesin-bound region. **G)** Bar plot of the percentage of BL6-specific sites (subspecies-specific and the tissue-shared subset) present within each signal tier from (**A**), from the total set of CTCF sites of that type.

**Additional file 5: Figure S5.** Chromosome 4 Interferon-zeta gene-cluster shows negligible transcriptional activity, and weak 3D interaction. **Top)** 10 kb resolution Hi-C heatmap of 3D genomic interactions in the 200 kb locus on Chromosome 4 comprising the Interferon-zeta gene-cluster (57.57 kb) and its genomic neighbourhood, generated using The 3D Genome Browser [67] based on Bonev et al. [54]. The shaded area (*grey*) denotes the genomic window of the Interferon-zeta gene-cluster, showing weak long-range interaction encompassing the cluster, albeit in the intergenic distance between two neighbouring TADs. The units on the scale (*top left*) are for normalised interacting counts. **Bottom)** The first two tracks show the RNA-seq alignments for all available tissues from the Ensembl browser [63] (*top*) and liver (*bottom*) for the entire 200 kb locus. The y axis denotes the number of RNA-seq reads mapping to each gene. All genes in the 200 kb window are denoted below, with arrowheads indicating the direction of transcription.

**Additional file 6: Figure S6.** Convergent evolution of an orthologous interferon gene cluster in pig. **A)** Genome browser display of BL6-Pig LASTz pairwise alignment of the 15-gene cluster. Pink tracks show the BL6 genome regions aligning to sequences in the pig genome. **B)** A zoom-in view of the orthologous gene cluster of interferon precursors in the pig genome. The orthologous gene in (A) is shown as the leftmost gene in the window in brown italics. The 12 paralogues to this gene are highlighted in brown italics with the other interferon genes in light grey. The arrowheads indicate the direction of transcription. The dark grey tracks at the bottom indicate the LTR repeat content of the region. **C)** A schematic diagram showing the position of the CTCF motif enriched at around 1 kb from the TSS of 12/13 genes in the cluster, with the motif composition below the orange track indicating the position. The motif underneath is the CTCF canonical motif with the *p*-value of the probability that the match occurred by random chance.

## Data Availability

All mouse ChIP-seq libraries utilised in this study are readily available through the Array Express repository (https://www.ebi.ac.uk/arrayexpress/) under these accession numbers: E-MTAB-5769 (for BL6) [37], E-MTAB-8014 (for CAST) [44], E-MTAB-941 (Cohesin) [55], E-MTAB-1414 (FOXA1 and CEBPA) [47], E-MTAB-4089 (H3K4me3 and H3K27ac) [75], E-MTAB-1091 (RNA-seq) [52]. The TAD data used in the analysis were obtained from Vietri Rudan et al. [29] and Bonev et al. [54]. ENCODE data was used for CTCF binding across tissues [48]. Selected scripts and pipelines are available from this GitHub repository: https://github.com/Dhoyazan/Functional-signatures-of-evolutionarily-young-CTCF-binding-sites

## References

[1] ENCODE Project Consortium (2007). Identification and analysis of functional elements in 1% of the human genome by the ENCODE pilot project. Nature.

[2] Vierstra J, Rynes E, Sandstrom R, Zhang M, Canfield T, Hansen RS, Stehling-Sun S, Sabo PJ, Byron R, Humbert R, Thurman RE, Johnson AK, Vong S, Lee K, Bates D, Neri F, Diegel M, Giste E, Haugen E, Dunn D, Wilken MS, Josefowicz S, Samstein R, Chang KH, Eichler EE, De Bruijn M, Reh TA, Skoultchi A, Rudensky A, Orkin SH, Papayannopoulou T, Treuting PM, Selleri L, Kaul R, Groudine M, Bender MA, Stamatoyannopoulos JA (2014). Mouse regulatory DNA landscapes reveal global principles of cis-regulatory evolution. Science.

[3] Wang K, Li M, Hakonarson H (2010). Analysing biological pathways in genome-wide association studies. Nat Rev Genet.

[4] Cooper GM, Brudno M, Stone EA, Dubchak I, Batzoglou S, Sidow A (2004). Characterization of evolutionary rates and constraints in three mammalian genomes. Genome Res.

[5] Carvunis AR, Wang T, Skola D, Yu A, Chen J, Kreisberg JF, Ideker T. Evidence for a common evolutionary rate in metazoan transcriptional networks. Elife. 2015;4:e11615. 10.7554/eLife.11615 PMC4764585 26682651

[6] Dermitzakis ET, Clark AG (2002). Evolution of transcription factor binding sites in mammalian gene regulatory regions: conservation and turnover. Mol Biol Evol.

[7] Schmidt D, Wilson MD, Ballester B, Schwalie PC, Brown GD, Marshall A, Kutter C, Watt S, Martinez-Jimenez CP, Mackay S, Talianidis I, Flicek P, Odom DT (2010). Five-vertebrate ChIP-seq reveals the evolutionary dynamics of transcription factor binding. Science.

[8] Xiao S, Xie D, Cao X, Yu P, Xing X, Chen CC, Musselman M, Xie M, West FD, Lewin HA, Wang T, Zhong S (2012). Comparative epigenomic annotation of regulatory DNA. Cell.

[9] Phillips JE, Corces VG (2009). CTCF: master weaver of the genome. Cell.

[10] Klenova EM, Nicolas RH, Paterson HF, Carne AF, Heath CM, Goodwin GH, Neiman PE, Lobanenkov VV (1993). CTCF, a conserved nuclear factor required for optimal transcriptional activity of the chicken c-myc gene, is an 11-Zn-finger protein differentially expressed in multiple forms. Mol Cell Biol.

[11] Hadjur S, Williams LM, Ryan NK, Cobb BS, Sexton T, Fraser P, Fisher AG, Merkenschlager M. Cohesins form chromosomal cis-interactions at the developmentally regulated IFNG locus. Nature. 2009;460:410–3. 10.1038/nature08079 PMC2869028 19458616

[12] Splinter E, Heath H, Kooren J, Palstra RJ, Klous P, Grosveld F, Galjart N, de Laat W (2006). CTCF mediates long-range chromatin looping and local histone modification in the beta-globin locus. Genes Dev.

[13] Nora EP, Lajoie BR, Schulz EG, Giorgetti L, Okamoto I, Servant N, Piolot T, van Berkum NL, Meisig J, Sedat J, Gribnau J, Barillot E, Blüthgen N, Dekker J, Heard E. Spatial partitioning of the regulatory landscape of the X-inactivation centre. Nature. 2012; 485, 381–385. 10.1038/nature11049 PMC3555144 22495304

[14] Stadhouders R, Thongjuea S, Andrieu-Soler C, Palstra RJ, Bryne JC, van den Heuvel A, Stevens M, de Boer E, Kockx C, van der Sloot A, van den Hout M, van Ijcken W, Eick D, Lenhard B, Grosveld F, Soler E (2012). Dynamic long-range chromatin interactions control Myb proto-oncogene transcription during erythroid development. EMBO J.

[15] Xie X, Mikkelsen TS, Gnirke A, Lindblad-Toh K, Kellis M, Lander ES (2007). Systematic discovery of regulatory motifs in conserved regions of the human genome, including thousands of CTCF insulator sites. Proc Natl Acad Sci U S A.

[16] Ruiz-Velasco M, Kumar M, Lai MC, Bhat P, Solis-Pinson AB, Reyes A, Kleinsorg S, Noh KM, Gibson TJ, Zaugg JB (2017). CTCF-mediated chromatin loops between promoter and gene body regulate alternative splicing across individuals. Cell Syst.

[17] Fedoriw AM, Stein P, Svoboda P, Schultz RM, Bartolomei MS (2004). Transgenic RNAi reveals essential function for CTCF in H19 gene imprinting. Science.

[18] Heath H, de Almeida CR, Sleutels F, Dingjan G, van de Nobelen S, Jonkers I, Ling KW, Gribnau J, Renkawitz R, Grosveld F, Hendriks RW, Galjart N (2008). CTCF regulates cell cycle progression of alphabeta T cells in the thymus. EMBO J.

[19] Soshnikova N, Montavon T, Leleu M, Galjart N, Duboule D (2010). Functional analysis of CTCF during mammalian limb development. Dev Cell.

[20] Filippova GN, Fagerlie S, Klenova EM, Myers C, Dehner Y, Goodwin G, Neiman PE, Collins SJ, Lobanenkov VV (1996). An exceptionally conserved transcriptional repressor, CTCF, employs different combinations of zinc fingers to bind diverged promoter sequences of avian and mammalian c-myc oncogenes. Mol Cell Biol.

[21] Pugacheva EM, Kwon YW, Hukriede NA, Pack S, Flanagan PT, Ahn JC, Park JA, Choi KS, Kim KW, Loukinov D, Dawid IB, Lobanenkov VV (2006). Cloning and characterization of zebrafish CTCF: developmental expression patterns, regulation of the promoter region, and evolutionary aspects of gene organization. Gene.

[22] Hnisz D, Weintraub AS, Day DS, Valton AL, Bak RO, Li CH, Goldmann J, Lajoie BR, Fan ZP, Sigova AA, Reddy J, Borges-Rivera D, Lee TI, Jaenisch R, Porteus MH, Dekker J, Young RA (2016). Activation of proto-oncogenes by disruption of chromosome neighborhoods. Science.

[23] Merkenschlager M, Odom DT (2013). CTCF and cohesin: linking gene regulatory elements with their targets. Cell.

[24] Sofueva S, Yaffe E, Chan WC, Georgopoulou D, Vietri Rudan M, Mira-Bontenbal H, Pollard SM, Schroth GP, Tanay A, Hadjur S (2013). Cohesin-mediated interactions organize chromosomal domain architecture. EMBO J.

[25] Zuin J, Dixon JR, van der Reijden MI, Ye Z, Kolovos P, Brouwer RW, van de Corput MP, van de Werken HJ, Knoch TA, van IJcken WF, Grosveld FG, Ren B, Wendt KS (2014). Cohesin and CTCF differentially affect chromatin architecture and gene expression in human cells. Proc Natl Acad Sci U S A.

[26] Ji X, Dadon DB, Powell BE, Fan ZP, Borges-Rivera D, Shachar S, Weintraub AS, Hnisz D, Pegoraro G, Lee TI, Misteli T, Jaenisch R, Young RA (2016). 3D chromosome regulatory landscape of human pluripotent cells. Cell Stem Cell.

[27] Ong CT, Corces VG (2014). CTCF: an architectural protein bridging genome topology and function. Nat Rev Genet.

[28] Sanborn AL, Rao SS, Huang SC, Durand NC, Huntley MH, Jewett AI, Bochkov ID, Chinnappan D, Cutkosky A, Li J, Geeting KP, Gnirke A, Melnikov A, McKenna D, Stamenova EK, Lander ES, Aiden EL (2015). Chromatin extrusion explains key features of loop and domain formation in wild-type and engineered genomes. Proc Natl Acad Sci U S A.

[29] Vietri Rudan M, Barrington C, Henderson S, Ernst C, Odom DT, Tanay A, Hadjur S (2015). Comparative hi-C reveals that CTCF underlies evolution of chromosomal domain architecture. Cell Rep.

[30] Dixon JR, Selvaraj S, Yue F, Kim A, Li Y, Shen Y, Hu M, Liu JS, Ren B (2012). Topological domains in mammalian genomes identified by analysis of chromatin interactions. Nature.

[31] Harmston N, Ing-Simmons E, Tan G, Perry M, Merkenschlager M, Lenhard B (2017). Topologically associating domains are ancient features that coincide with metazoan clusters of extreme noncoding conservation. Nat Commun.

[32] Bourque G, Leong B, Vega VB, Chen X, Lee YL, Srinivasan KG, Chew J-L, Ruan Y, Wei C-L, Ng HH, Liu ET (2008). Evolution of the mammalian transcription factor binding repertoire via transposable elements. Genome Res.

[33] Feschotte C (2008). Transposable elements and the evolution of regulatory networks. Nat Rev Genet.

[34] Kunarso G, Chia N-Y, Jeyakani J, Hwang C, Lu X, Chan Y-S, Ng H-H, Bourque G (2010). Transposable elements have rewired the core regulatory network of human embryonic stem cells. Nat Genet.

[35] Schmidt D, Schwalie PC, Wilson MD, Ballester B, Gonçalves A, Kutter C, Brown GD, Marshall A, Flicek P, Odom DT (2012). Waves of retrotransposon expansion remodel genome organization and CTCF binding in multiple mammalian lineages. Cell.

[36] Sundaram V, Cheng Y, Ma Z, Li D, Xing X, Edge P, Snyder MP, Wang T (2014). Widespread contribution of transposable elements to the innovation of gene regulatory networks. Genome Res.

[37] Thybert D, Roller M, Navarro FCP, Fiddes I, Streeter I, Feig C, Martin-Galvez D, Kolmogorov M, Janoušek V, Akanni W, Aken B, Aldridge S, Chakrapani V, Chow W, Clarke L, Cummins C, Doran A, Dunn M, Goodstadt L, Howe K, Howell M, Josselin AA, Karn RC, Laukaitis CM, Jingtao L, Martin F, Muffato M, Nachtweide S, Quail MA, Sisu C, Stanke M, Stefflova K, Van Oosterhout C, Veyrunes F, Ward B, Yang F, Yazdanifar G, Zadissa A, Adams DJ, Brazma A, Gerstein M, Paten B, Pham S, Keane TM, Odom DT, Flicek P (2018). Repeat associated mechanisms of genome evolution and function revealed by the *Mus caroli* and *Mus pahari* genomes. Genome Res.

[38] Ward MC, Wilson MD, Barbosa-Morais NL, Schmidt D, Stark R, Pan Q, Schwalie PC, Menon S, Lukk M, Watt S, Thybert D, Kutter C, Kirschner K, Flicek P, Blencowe BJ, Odom DT (2013). Latent regulatory potential of human-specific repetitive elements. Mol Cell.

[39] Lynch VJ, Leclerc RD, May G, Wagner GP (2011). Transposon-mediated rewiring of gene regulatory networks contributed to the evolution of pregnancy in mammals. Nat Genet.

[40] Doran AG, Wong K, Flint J, Adams DJ, Hunter KW, Keane TM (2016). Deep genome sequencing and variation analysis of 13 inbred mouse strains defines candidate phenotypic alleles, private variation and homozygous truncating mutations. Genome Biol.

[41] Keane TM, Goodstadt L, Danecek P, White MA, Wong K, Yalcin B, Heger A, Agam A, Slater G, Goodson M, Furlotte NA, Eskin E, Nellåker C, Whitley H, Cleak J, Janowitz D, Hernandez-Pliego P, Edwards A, Belgard TG, Oliver PL, RE MI, Bhomra A, Nicod J, Gan X, Yuan W, van der Weyden L, Steward CA, Bala S, Stalker J, Mott R, Durbin R, Jackson IJ, Czechanski A, Guerra-Assunção JA, Donahue LR, Reinholdt LG, Payseur BA, Ponting CP, Birney E, Flint J, Adams DJ (2011). Mouse genomic variation and its effect on phenotypes and gene regulation. Nature.

[42] Lilue J, Doran AG, Fiddes IT, Abrudan M, Armstrong J, Bennett R, Chow W, Collins J, Collins S, Czechanski A, Danecek P, Diekhans M, Dolle DD, Dunn M, Durbin R, Earl D, Ferguson-Smith A, Flicek P, Flint J, Frankish A, Fu B, Gerstein M, Gilbert J, Goodstadt L, Harrow J, Howe K, Ibarra-Soria X, Kolmogorov M, Lelliott CJ, Logan DW, Loveland J, Mathews CE, Mott R, Muir P, Nachtweide S, FCP N, Odom DT, Park N, Pelan S, Pham SK, Quail M, Reinholdt L, Romoth L, Shirley L, Sisu C, Sjoberg-Herrera M, Stanke M, Steward C, Thomas M, Threadgold G, Thybert D, Torrance J, Wong K, Wood J, Yalcin B, Yang F, Adams DJ, Paten B, Keane TM (2018). Sixteen diverse laboratory mouse reference genomes define strain-specific haplotypes and novel functional loci. Nat Genet.

[43] Mouse Genome Sequencing Consortium (2002). Initial sequencing and comparative analysis of the mouse genome. Nature.

[44] Kentepozidou E, Aitken SJ, Feig C, Stefflova K, Ibarra-Soria X, Odom DT, Roller M, Flicek P (2020). Clustered CTCF binding is an evolutionary mechanism to maintain topologically associating domains. Genome Biol.

[45] Holwerda SJ, de Laat W (2013). CTCF: the protein, the binding partners, the binding sites and their chromatin loops. Philos Trans R Soc Lond Ser B Biol Sci.

[46] Hickey G, Paten B, Earl D, Zerbino D, Haussler D (2013). HAL: a hierarchical format for storing and analyzing multiple genome alignments. Bioinformatics.

[47] Stefflova K, Thybert D, Wilson MD, Streeter I, Aleksic J, Karagianni P, Brazma A, Adams DJ, Talianidis I, Marioni JC, Flicek P, Odom DT (2013). Cooperativity and rapid evolution of cobound transcription factors in closely related mammals. Cell.

[48] Yue F, Cheng Y, Breschi A, Vierstra J, Wu W, Ryba T, Sandstrom R, Ma Z, Davis C, Pope BD, Shen Y, Pervouchine DD, Djebali S, Thurman RE, Kaul R, Rynes E, Kirilusha A, Marinov GK, Williams BA, Trout D, Amrhein H, Fisher-Aylor K, Antoshechkin I, DeSalvo G, See L-H, Fastuca M, Drenkow J, Zaleski C, Dobin A, Prieto P, Lagarde J, Bussotti G, Tanzer A, Denas O, Li K, Bender MA, Zhang M, Byron R, Groudine MT, McCleary D, Pham L, Ye Z, Kuan S, Edsall L, Wu Y-C, Rasmussen MD, Bansal MS, Kellis M, Keller CA, Morrissey CS, Mishra T, Jain D, Dogan N, Harris RS, Cayting P, Kawli T, Boyle AP, Euskirchen G, Kundaje A, Lin S, Lin Y, Jansen C, Malladi VS, Cline MS, Erickson DT, Kirkup VM, Learned K, Sloan CA, Rosenbloom KR, Lacerda de Sousa B, Beal K, Pignatelli M, Flicek P, Lian J, Kahveci T, Lee D, Kent WJ, Ramalho Santos M, Herrero J, Notredame C, Johnson A, Vong S, Lee K, Bates D, Neri F, Diegel M, Canfield T, Sabo PJ, Wilken MS, Reh TA, Giste E, Shafer A, Kutyavin T, Haugen E, Dunn D, Reynolds AP, Neph S, Humbert R, Hansen RS, De Bruijn M, Selleri L, Rudensky A, Josefowicz S, Samstein R, Eichler EE, Orkin SH, Levasseur D, Papayannopoulou T, Chang K-H, Skoultchi A, Gosh S, Disteche C, Treuting P, Wang Y, Weiss MJ, Blobel GA, Cao X, Zhong S, Wang T, Good PJ, Lowdon RF, Adams LB, Zhou X-Q, Pazin MJ, Feingold EA, Wold B, Taylor J, Mortazavi A, Weissman SM, Stamatoyannopoulos JA, Snyder MP, Guigo R, Gingeras TR, Gilbert DM, Hardison RC, Beer MA, Ren B, Consortium MENCODE (2014). A comparative encyclopedia of DNA elements in the mouse genome. Nature.

[49] Glinsky GV (2018). Contribution of transposable elements and distal enhancers to evolution of human-specific features of interphase chromatin architecture in embryonic stem cells. Chromosom Res.

[50] Trizzino M, Kapusta A, Brown CD (2018). Transposable elements generate regulatory novelty in a tissue-specific fashion. BMC Genomics.

[51] Shannon CE (1948). A mathematical theory of communication. Bell Syst Tech J.

[52] Goncalves A, Leigh-Brown S, Thybert D, Stefflova K, Turro E, Flicek P, Brazma A, Odom DT, Marioni JC (2012). Extensive compensatory cis-trans regulation in the evolution of mouse gene expression. Genome Res.

[53] Ziebarth JD, Bhattacharya A, Cui Y (2013). CTCFBSDB 2.0: a database for CTCF-binding sites and genome organization. Nucleic Acids Res.

[54] Bonev B, Mendelson Cohen N, Szabo Q, Fritsch L, Papadopoulos GL, Lubling Y, Xu X, Lv X, Hugnot JP, Tanay A, Cavalli G (2017). Multiscale 3D genome rewiring during mouse neural development. Cell.

[55] Faure AJ, Schmidt D, Watt S, Schwalie PC, Wilson MD, Xu H, Ramsay RG, Odom DT, Flicek P (2012). Cohesin regulates tissue-specific expression by stabilizing highly occupied cis-regulatory modules. Genome Res.

[56] Casa V, Moronta Gines M, Gade Gusmao E, Slotman JA, Zirkel A, Josipovic N, Oole E, van IJcken WFJ, Houtsmuller AB, Papantonis A, Wendt KS. Redundant and specific roles of cohesin STAG subunits in chromatin looping and transcription control. Genome Res. 2020. 10.1101/gr.253211.119. 10.1101/gr.253211.119 PMC7197483 32253279

[57] Kojic A, Cuadrado A, De Koninck M, Giménez-Llorente D, Rodríguez-Corsino M, Gómez-López G, Le Dily F, Marti-Renom MA, Losada A (2018). Distinct roles of cohesin-SA1 and cohesin-SA2 in 3D chromosome organization. Nat Struct Mol Biol.

[58] Frankish A, Diekhans M, Ferreira AM, Johnson R, Jungreis I, Loveland J, Mudge JM, Sisu C, Wright J, Armstrong J, Barnes I, Berry A, Bignell A, Carbonell Sala S, Chrast J, Cunningham F, Di Domenico T, Donaldson S, Fiddes IT, García Girón C, Gonzalez JM, Grego T, Hardy M, Hourlier T, Hunt T, Izuogu OG, Lagarde J, Martin FJ, Martínez L, Mohanan S, Muir P, FCP N, Parker A, Pei B, Pozo F, Ruffier M, Schmitt BM, Stapleton E, Suner MM, Sycheva I, Uszczynska-Ratajczak B, Xu J, Yates A, Zerbino D, Zhang Y, Aken B, Choudhary JS, Gerstein M, Guigó R, TJP H, Kellis M, Paten B, Reymond A, Tress ML, Flicek P (2019). GENCODE reference annotation for the human and mouse genomes. Nucleic Acids Res.

[59] Kawai J, Shinagawa A, Shibata K, Yoshino M, Itoh M, Ishii Y, Arakawa T, Hara A, Fukunishi Y, Konno H, Adachi J, Fukuda S, Aizawa K, Izawa M, Nishi K, Kiyosawa H, Kondo S, Yamanaka I, Saito T, Okazaki Y, Gojobori T, Bono H, Kasukawa T, Saito R, Kadota K, Matsuda H, Ashburner M, Batalov S, Casavant T, Fleischmann W, Gaasterland T, Gissi C, King B, Kochiwa H, Kuehl P, Lewis S, Matsuo Y, Nikaido I, Pesole G, Quackenbush J, Schriml LM, Staubli F, Suzuki R, Tomita M, Wagner L, Washio T, Sakai K, Okido T, Furuno M, Aono H, Baldarelli R, Barsh G, Blake J, Boffelli D, Bojunga N, Carninci P, de Bonaldo MF, Brownstein MJ, Bult C, Fletcher C, Fujita M, Gariboldi M, Gustincich S, Hill D, Hofmann M, Hume DA, Kamiya M, Lee NH, Lyons P, Marchionni L, Mashima J, Mazzarelli J, Mombaerts P, Nordone P, Ring B, Ringwald M, Rodriguez I, Sakamoto N, Sasaki H, Sato K, Schönbach C, Seya T, Shibata Y, Storch KF, Suzuki H, Toyo-oka K, Wang KH, Weitz C, Whittaker C, Wilming L, Wynshaw-Boris A, Yoshida K, Hasegawa Y, Kawaji H, Kohtsuki S, Hayashizaki Y, RIKEN GERGPIITATFANTOMC (2001). Functional annotation of a full-length mouse cDNA collection. Nature.

[60] Okazaki Y, Furuno M, Kasukawa T, Adachi J, Bono H, Kondo S, Nikaido I, Osato N, Saito R, Suzuki H, Yamanaka I, Kiyosawa H, Yagi K, Tomaru Y, Hasegawa Y, Nogami A, Schönbach C, Gojobori T, Baldarelli R, Hill DP, Bult C, Hume DA, Quackenbush J, Schriml LM, Kanapin A, Matsuda H, Batalov S, Beisel KW, Blake JA, Bradt D, Brusic V, Chothia C, Corbani LE, Cousins S, Dalla E, Dragani TA, Fletcher CF, Forrest A, Frazer KS, Gaasterland T, Gariboldi M, Gissi C, Godzik A, Gough J, Grimmond S, Gustincich S, Hirokawa N, Jackson IJ, Jarvis ED, Kanai A, Kawaji H, Kawasawa Y, Kedzierski RM, King BL, Konagaya A, Kurochkin IV, Lee Y, Lenhard B, Lyons PA, Maglott DR, Maltais L, Marchionni L, McKenzie L, Miki H, Nagashima T, Numata K, Okido T, Pavan WJ, Pertea G, Pesole G, Petrovsky N, Pillai R, Pontius JU, Qi D, Ramachandran S, Ravasi T, Reed JC, Reed DJ, Reid J, Ring BZ, Ringwald M, Sandelin A, Schneider C, Semple CA, Setou M, Shimada K, Sultana R, Takenaka Y, Taylor MS, Teasdale RD, Tomita M, Verardo R, Wagner L, Wahlestedt C, Wang Y, Watanabe Y, Wells C, Wilming LG, Wynshaw-Boris A, Yanagisawa M, Yang I, Yang L, Yuan Z, Zavolan M, Zhu Y, Zimmer A, Carninci P, Hayatsu N, Hirozane-Kishikawa T, Konno H, Nakamura M, Sakazume N, Sato K, Shiraki T, Waki K, Kawai J, Aizawa K, Arakawa T, Fukuda S, Hara A, Hashizume W, Imotani K, Ishii Y, Itoh M, Kagawa I, Miyazaki A, Sakai K, Sasaki D, Shibata K, Shinagawa A, Yasunishi A, Yoshino M, Waterston R, Lander ES, Rogers J, Birney E, Hayashizaki Y, FANTOM C, RIKEN GERGPIIIT (2002). Analysis of the mouse transcriptome based on functional annotation of 60,770 full-length cDNAs. Nature.

[61] Oritani K, Medina KL, Tomiyama Y, Ishikawa J, Okajima Y, Ogawa M, Yokota T, Aoyama K, Takahashi I, Kincade PW, Matsuzawa Y (2000). Limitin: an interferon-like cytokine that preferentially influences B-lymphocyte precursors. Nat Med.

[62] Xu L, Yang L, Liu W (2013). Distinct evolution process among type I interferon in mammals. Protein Cell.

[63] Cunningham F, Achuthan P, Akanni W, Allen J, Amode MR, Armean IM, Bennett R, Bhai J, Billis K, Boddu S, Cummins C, Davidson C, Dodiya KJ, Gall A, Girón CG, Gil L, Grego T, Haggerty L, Haskell E, Hourlier T, Izuogu OG, Janacek SH, Juettemann T, Kay M, Laird MR, Lavidas I, Liu Z, Loveland JE, Marugán JC, Maurel T, McMahon AC, Moore B, Morales J, Mudge JM, Nuhn M, Ogeh D, Parker A, Parton A, Patricio M, Abdul Salam AI, Schmitt BM, Schuilenburg H, Sheppard D, Sparrow H, Stapleton E, Szuba M, Taylor K, Threadgold G, Thormann A, Vullo A, Walts B, Winterbottom A, Zadissa A, Chakiachvili M, Frankish A, Hunt SE, Kostadima M, Langridge N, Martin FJ, Muffato M, Perry E, Ruffier M, Staines DM, Trevanion SJ, Aken BL, Yates AD, Zerbino DR, Flicek P (2019). Ensembl 2019. Nucleic Acids Res.

[64] Janoušek V, Laukaitis CM, Yanchukov A, Karn RC (2016). The role of retrotransposons in gene family expansions in the human and mouse genomes. Genome Biol Evol.

[65] Creyghton MP, Cheng AW, Welstead GG, Kooistra T, Carey BW, Steine EJ, Hanna J, Lodato MA, Frampton GM, Sharp PA, Boyer LA, Young RA, Jaenisch R (2010). Histone H3K27ac separates active from poised enhancers and predicts developmental state. Proc Natl Acad Sci U S A.

[66] Schneider R, Bannister AJ, Myers FA, Thorne AW, Crane-Robinson C, Kouzarides T (2004). Histone H3 lysine 4 methylation patterns in higher eukaryotic genes. Nat Cell Biol.

[67] Wang Y, Song F, Zhang B, Zhang L, Xu J, Kuang D, Li D, Choudhary MNK, Li Y, Hu M, Hardison R, Wang T, Yue F (2018). The 3D genome browser: a web-based browser for visualizing 3D genome organization and long-range chromatin interactions. Genome Biol.

[68] Groenen MA, Archibald AL, Uenishi H, Tuggle CK, Takeuchi Y, Rothschild MF, Rogel-Gaillard C, Park C, Milan D, Megens HJ, Li S, Larkin DM, Kim H, Frantz LA, Caccamo M, Ahn H, Aken BL, Anselmo A, Anthon C, Auvil L, Badaoui B, Beattie CW, Bendixen C, Berman D, Blecha F, Blomberg J, Bolund L, Bosse M, Botti S, Bujie Z, Bystrom M, Capitanu B, Carvalho-Silva D, Chardon P, Chen C, Cheng R, Choi SH, Chow W, Clark RC, Clee C, Crooijmans RP, Dawson HD, Dehais P, De Sapio F, Dibbits B, Drou N, Du ZQ, Eversole K, Fadista J, Fairley S, Faraut T, Faulkner GJ, Fowler KE, Fredholm M, Fritz E, Gilbert JG, Giuffra E, Gorodkin J, Griffin DK, Harrow JL, Hayward A, Howe K, Hu ZL, Humphray SJ, Hunt T, Hornshøj H, Jeon JT, Jern P, Jones M, Jurka J, Kanamori H, Kapetanovic R, Kim J, Kim JH, Kim KW, Kim TH, Larson G, Lee K, Lee KT, Leggett R, Lewin HA, Li Y, Liu W, Loveland JE, Lu Y, Lunney JK, Ma J, Madsen O, Mann K, Matthews L, McLaren S, Morozumi T, Murtaugh MP, Narayan J, Nguyen DT, Ni P, Oh SJ, Onteru S, Panitz F, Park EW, Park HS, Pascal G, Paudel Y, Perez-Enciso M, Ramirez-Gonzalez R, Reecy JM, Rodriguez-Zas S, Rohrer GA, Rund L, Sang Y, Schachtschneider K, Schraiber JG, Schwartz J, Scobie L, Scott C, Searle S, Servin B, Southey BR, Sperber G, Stadler P, Sweedler JV, Tafer H, Thomsen B, Wali R, Wang J, Wang J, White S, Xu X, Yerle M, Zhang G, Zhang J, Zhang J, Zhao S, Rogers J, Churcher C, Schook LB (2012). Analyses of pig genomes provide insight into porcine demography and evolution. Nature.

[69] Román AC, González-Rico FJ, Moltó E, Hernando H, Neto A, Vicente-Garcia C, Ballestar E, Gómez-Skarmeta JL, Vavrova-Anderson J, White RJ, Montoliu L, Fernández-Salguero PM (2011). Dioxin receptor and SLUG transcription factors regulate the insulator activity of B1 SINE retrotransposons via an RNA polymerase switch. Genome Res.

[70] Yokoyama KD, Zhang Y, Ma J (2014). Tracing the evolution of lineage-specific transcription factor binding sites in a birth-death framework. PLoS Comput Biol.

[71] Chen H, Tian Y, Shu W, Bo X, Wang S (2012). Comprehensive identification and annotation of cell type-specific and ubiquitous CTCF-binding sites in the human genome. PLoS One.

[72] van Pesch V, Lanaya H, Renauld JC, Michiels T (2004). Characterization of the murine alpha interferon gene family. J Virol.

[73] Janoušek V, Karn RC, Laukaitis CM (2013). The role of retrotransposons in gene family expansions: insights from the mouse Abp gene family. BMC Evol Biol.

[74] Hardy MP, Owczarek CM, Jermiin LS, Ejdebäck M, Hertzog PJ (2004). Characterization of the type I interferon locus and identification of novel genes. Genomics.

[75] Wong ES, Schmitt BM, Kazachenka A, Thybert D, Redmond A, Connor F, Rayner TF, Feig C, Ferguson-Smith AC, Marioni JC, Odom DT, Flicek P (2017). Interplay of cis and trans mechanisms driving transcription factor binding and gene expression evolution. Nat Commun.

[76] Andrews S (2010). FastQC: a quality control tool for high throughput sequence data.

[77] Li H, Durbin R (2009). Fast and accurate short read alignment with Burrows-Wheeler transform. Bioinformatics.

[78] Li H, Handsaker B, Wysoker A, Fennell T, Ruan J, Homer N, Marth G, Abecasis G, Durbin R, 1000 Genome Project Data Processing Subgroup (2009). The sequence alignment/map format and SAMtools. Bioinformatics.

[79] Zhang Y, Liu T, Meyer CA, Eeckhoute J, Johnson DS, Bernstein BE, Nussbaum C, Myers RM, Brown M, Li W, Liu XS (2008). Model-based analysis of ChIP-Seq (MACS). Genome Biol.

[80] Bailey TL, Boden M, Buske FA, Frith M, Grant CE, Clementi L, Ren J, Li WW, Noble WS (2009). MEME SUITE: tools for motif discovery and searching. Nucleic Acids Res.

[81] Bailey TL, Johnson J, Grant CE, Noble WS (2015). The MEME suite. Nucleic Acids Res.

[82] Heinz S, Benner C, Spann N, Bertolino E, Lin YC, Laslo P, Cheng JX, Murre C, Singh H, Glass CK (2010). Simple combinations of lineage-determining transcription factors prime cis-regulatory elements required for macrophage and B cell identities. Mol Cell.

[83] Smit AFA, Hubley R, Green P (2013). RepeatMasker Open-4.0.

[84] Quinlan AR, Hall IM (2010). BEDTools: a flexible suite of utilities for comparing genomic features. Bioinformatics.

[85] Quinlan AR (2014). BEDTools: the Swiss-Army tool for genome feature analysis. Curr Protoc Bioinformatics.

[86] Gu Z, Eils R, Schlesner M (2016). Complex heatmaps reveal patterns and correlations in multidimensional genomic data. Bioinformatics.

[87] Oksanen J, Blanchet FG, Friendly M, Kindt R, Legendre P, McGlinn D, Minchin PR, O’Hara RB, Simpson GL, Solymos P, Stevens MHH, Szoecs E, Wagner H (2019). vegan: Community Ecology Package.

[88] McLean CY, Bristor D, Hiller M, Clarke SL, Schaar BT, Lowe CB, Wenger AM, Bejerano G (2010). GREAT improves functional interpretation of cis-regulatory regions. Nat Biotechnol.

[89] Dobin A, Davis CA, Schlesinger F, Drenkow J, Zaleski C, Jha S, Batut P, Chaisson M, Gingeras TR (2013). STAR: ultrafast universal RNA-seq aligner. Bioinformatics.

[90] Anders S, Pyl PT, Huber W (2015). HTSeq--a Python framework to work with high-throughput sequencing data. Bioinformatics.

[91] Love MI, Huber W, Anders S (2014). Moderated estimation of fold change and dispersion for RNA-seq data with DESeq2. Genome Biol.

[92] Ashburner M, Ball CA, Blake JA, Botstein D, Butler H, Cherry JM, Davis AP, Dolinski K, Dwight SS, Eppig JT, Harris MA, Hill DP, Issel-Tarver L, Kasarskis A, Lewis S, Matese JC, Richardson JE, Ringwald M, Rubin GM, Sherlock G (2000). Gene ontology: tool for the unification of biology. The Gene Ontology Consortium. Nat Genet.

[93] Mi H, Muruganujan A, Ebert D, Huang X, Thomas PD (2019). PANTHER version 14: more genomes, a new PANTHER GO-slim and improvements in enrichment analysis tools. Nucleic Acids Res.

[94] The Gene Ontology Consortium (2019). The gene ontology resource: 20 years and still GOing strong. Nucleic Acids Res.

[95] Ambrosini G, Groux R, Bucher P (2018). PWMScan: a fast tool for scanning entire genomes with a position-specific weight matrix. Bioinformatics.

[96] Neph S, Kuehn MS, Reynolds AP, Haugen E, Thurman RE, Johnson AK, Rynes E, Maurano MT, Vierstra J, Thomas S, Sandstrom R, Humbert R, Stamatoyannopoulos JA (2012). BEDOPS: high-performance genomic feature operations. Bioinformatics.

[97] Sievers F, Wilm A, Dineen D, Gibson TJ, Karplus K, Li W, Lopez R, McWilliam H, Remmert M, Söding J, Thompson JD, Higgins DG (2011). Fast, scalable generation of high-quality protein multiple sequence alignments using Clustal omega. Mol Syst Biol.

